# Atypical Resting-State EEG Graph Metrics of Network Efficiency Across Development in Autism and Their Association with Social Cognition: Results from the LEAP Study

**DOI:** 10.1007/s10803-025-06731-0

**Published:** 2025-02-14

**Authors:** E. de Jonge, P. Garcés, A. de Bildt, Y. Groen, E. J. H. Jones, L. Mason, R. J. Holt, H. Hayward, D. Murphy, B. Oakley, T. Charman, J. Ahmad, S. Baron-Cohen, M. H. Johnson, T. Banaschewski, S. Durston, B. Oranje, S. Bölte, J. Buitelaar, The EU-AIMS LEAP group, P. J. Hoekstra, A. Dietrich

**Affiliations:** 1 Department of Child & Adolescent Psychiatry & Accare Child Study Center, University of Groningen, University Medical Center Groningen, Lübeckweg 2, 9723 HE Groningen, The Netherlands; 2 Roche Pharma Research and Early Development, Neuroscience and Rare Diseases, Roche Innovation Center Basel, Grenzacherstrasse 124, CH-4070 Basel, Switzerland; 3 Department of Clinical and Developmental Neuropsychology, University of Groningen, Grote Kruisstraat 2/1, 9712 TS Groningen, The Netherlands; 4 Centre for Brain and Cognitive Development, Birkbeck, University of London, 32 Torrington Square, London, WC1E 7JL UK; 5 Department of Forensic and Neurodevelopmental Sciences, Institute of Psychiatry, Psychology and Neuroscience, King’s College London, De Crespigny Park, Denmark Hill, London, SE5 8AF UK; 6 Department of Psychiatry, Autism Research Centre, University of Cambridge, Douglas House, 18B Trumpington Road, Cambridge, CB2 8AH UK; 7 Department of Psychology, Institute of Psychiatry, Psychology and Neuroscience, King’s College London, De Crespigny Park, Denmark Hill, London, SE5 8AF UK; 8 School of Human Sciences, University of Greenwich, London, SE10 9LS England; 9 Centre for Brain & Cognitive Development, University of London, London, WC1E 7HX England; 10 Department of Psychology, University of Cambridge, Cambridge, CB2 3EB England; 11 Medical Faculty Mannheim, Heidelberg University, Central Institute of Mental Health, Mannheim, Germany; 12 Deptartment of Psychiatry, Brain Center, University Medical Center Utrecht, Utrecht, The Netherlands; 13 Center for Neuropsychiatric Schizophrenia Research (CNSR) and Center for Clinical Intervention and Neuropsychiatric Schizophrenia Research (CINS), Copenhagen University Hospital-Mental Health Services CPH, Glostrup, Denmark; 14 Department of Women’s and Children’s Health, Center of Neurodevelopmental Disorders (KIND), Centre for Psychiatry Research, Karolinska Institutet & Stockholm Health Care Services, Region Stockholm, Stockholm, Sweden; 15 Child and Adolescent Psychiatry, Stockholm Health Care Services, Region Stockholm, Stockholm, Sweden; 16 Curtin Autism Research Group, Curtin School of Allied Health, Curtin University, Perth, WA Australia; 17 Department of Cognitive Neuroscience, Donders Institute for Brain Cognition & Behavior, Radboud University Medical Center, Nijmegen, Gelderland The Netherlands; 18 Karakter Child & Adolescent Psychiatry University Centre, Nijmegen, Gelderland The Netherlands

**Keywords:** Autism spectrum disorder, EEG, Resting state, Graph theory, Social cognition

## Abstract

Autism has been associated with differences in functional brain network organization. However, the exact nature of these differences across development compared to non-autistic individuals and their relationship to autism-related social cognition, remains unclear. This study first aimed to identify EEG resting-state network characteristics in autistic versus non-autistic children, adolescents, and adults. Second, we investigated associations with social cognition measures. Analyzing resting-state EEG data from the EU-AIMS Longitudinal European Autism Project, we compared network metrics (global efficiency, clustering coefficient, and small-worldness) between 344 autistic and non-autistic individuals within and across age groups in four frequency bands (delta, theta, alpha, and beta). If significant, we explored their relationships to measures of empathy (empathy quotient), complex emotion recognition [reading the mind in the eyes task (RMET)], and theory of mind (animated shapes task). Compared to their non-autistic peers, autistic adolescents showed lower alpha global efficiency, while autistic adults showed lower alpha clustering and small-worldness. No network differences were observed among children. In adolescents, higher long-range integration was tentatively associated with higher RMET scores; in those with high autistic traits, higher long-range integration related to fewer parent-reported empathic behaviors. No brain-behavior relationships were observed in adults. Our findings suggest subtle differences in network topology between autistic and non-autistic individuals, with less efficient long-range efficiency during adolescence, and less local and overall network efficiency in adulthood. Furthermore, long-range integration may play a role in complex emotion recognition and empathy difficulties associated with autism in adolescence.

**Supplementary Information:**

The online version contains supplementary material available at 10.1007/s10803-025-06731-0.

## Introduction

Autism spectrum disorder (hereafter autism) is a neurodevelopmental condition associated with difficulties in social cognition, such as cognitive empathy (i.e., the ability to understand another’s mental state and to respond with a reciprocal emotion or action; Harmsen, 2019; Sucksmith et al., 2013), complex emotion recognition (Baron-Cohen et al., 2001; Oakley et al., 2016), and theory of mind (ToM; i.e., the ability to attribute mental states to oneself and others; Velikonja et al., 2019; Wilson, 2021). Research suggests that autism is related to atypical resting-state functional connectivity (i.e., synchrony of activity between brain regions; Belmonte et al., 2004; Just et al., 2004). Functional connectivity across and within brain regions is also critical for complex cognitive functions like social cognition, including empathy, complex emotion recognition, and ToM (Müller & Fishman, 2018; Van Overwalle, 2009). However, whether atypical resting-state network connectivity in autism contributes to its associated difficulties in social cognition remains understudied.

Where most studies on resting-state functional connectivity in autism have used functional magnetic resonance imaging (fMRI; for review see Lau et al., 2019), electroencephalography (EEG) offers several advantages. As opposed to fMRI, it directly measures neuronal activity, provides higher temporal resolution (allowing studying networks within different frequency bands), and is less invasive (greater ease of use with a diverse range of participants). EEG studies, utilizing a variety of connectivity measures, have generally indicated reduced long-range connectivity in autistic compared to non-autistic individuals across age, while differences in short-range connectivity have been less established (for review see O’Reilly et al., 2017). Currently, the exact nature of EEG network connectivity in autism remains incompletely understood, especially across different connectivity measures, frequency bands, and developmental stages (O’Reilly et al., 2017; for review on MRI and MEG see Picci et al., 2016).

A recent study on functional connectivity in autism using resting-state EEG data from the Longitudinal European Autism Project (LEAP) did not find autism-related differences as quantified by the weighted Phase Lag Index [wPLI; a measure of phase synchronization between EEG signals discarding zero-lag synchronization (Vinck et al., 2011)] in children, adolescents, or adults (Garcés et al., 2022). Yet, compared to overall connectivity measures, applying graph theory to resting-state EEG functional connectivity data allows for a more detailed quantification of functional network characteristics. It provides a mathematical framework to study complex networks and the information flow in them. By quantifying properties including network topology, efficiency, and organization, graph theory can facilitate a more comprehensive understanding of connectivity (Bullmore & Sporns, 2009; Rubinov & Sporns, 2010).

The few EEG resting state connectivity studies that have used graph theory to study autism have yielded inconsistent results so far. Some studies have reported lower network connectivity in autistic compared to non-autistic individuals through less efficient long-range network connectivity (as reflected by increased path length or decreased global efficiency), reduced local information processing (as reflected by decreased clustering coefficients), and/or architectures less typical of small-world networks (i.e., suboptimal equilibrium between long-range and local information processing) across autistic toddlers (Boersma et al., 2013), children (Zeng et al., 2017), and adults (Barttfeld et al., 2011). Moreover, in an adult neurotypical sample, autistic traits were associated with deviations from the optimal small-world network structure (Barttfeld et al., 2013). However, opposite patterns of decreased path length in autistic children (Zeng et al., 2017) and increased local connectivity in autistic children and young adults (Peters et al., 2013) have also been reported. Regarding neurotypical development, a further differentiation of long-range connectivity across age would be expected (Edde et al., 2021), however, patterns are currently unclear regarding graph metrics in autistic individuals as both under- and overconnectivity appear plausible (O’Reilly et al., 2017). Also, studies in adolescents are lacking as are studies simultaneously investigating multiple age periods (Picci et al., 2016). Moreover, the examined frequency bands varied between studies, likely giving rise to conflicting findings (Barttfeld et al., 2011; Boersma et al., 2013; Peters et al., 2013). This highlights the need to evaluate graph metrics across multiple frequency bands within the same study. Most importantly, however, sample sizes were generally small (ranging from 10 to 21 individuals with autism; Barttfeld et al., 2011; Boersma et al., 2013; Peters et al., 2013; Zeng et al., 2017), which may have biased the results. Given the heterogeneous nature of autism, larger samples are needed to be able to meaningfully capture this diversity. In sum, EEG resting-state graph metric studies in larger samples considering multiple frequency bands and developmental stages are needed. Addressing these elements together in one study using consistent methodologies will provide a more comprehensive view of brain connectivity in autism, which is difficult to achieve between studies with differing analysis techniques.

Additionally, these investigations could provide insight into context-independent markers of the social-cognitive characteristics associated with autism (Picci et al., 2016). While the EEG network properties have been linked to various cognitive domains, e.g., IQ (Zakharov et al., 2020), memory performance (Vecchio et al., 2016), and literacy skills (Lui et al., 2021), it remains unknown whether network atypicalities associated with autism are related to its social cognitive characteristics. To our knowledge, this is the first study to investigate the link between atypical resting-state EEG graph metrics and measures of empathy, complex emotion recognition, and ToM in autism.

The aim of the current paper was twofold: (1) to identify autism-related EEG resting-state characteristics of network efficiency across different developmental stages in a sizeable sample, and (2) to investigate their relationship with social cognition measures. We investigated differences between autistic and non-autistic individuals in three resting-state EEG network metrics that have previously been indicated in autism (path length/global efficiency, clustering, and small-worldness), across four canonical frequency bands (delta, theta, alpha, beta), and three age groups (children, adolescents, and adults), using data from the EU-AIMS LEAP study, a large multicenter initiative aimed at identifying biomarkers in autism (Charman et al., 2017; Loth et al., 2017). We also examined dimensional relationships between graph metrics and autistic trait scores across autistic and non-autistic participants (Constantino & Todd, 2003). We further investigated whether autism-related network structures were predictive of parent-reported empathy and performance on social cognition tests, in order to provide a more meaningful interpretation of autism-related connectivity patterns. As noted above, in light of changes in brain maturation across development, with typically stronger local efficiency (clustering) in childhood and increasing long-range and overall efficiency (global efficiency, small-worldness) across age (Edde et al., 2021), we expected developmental differences in autism-related connectivity suggesting delayed or atypical development. Also, we hypothesized lower network efficiency to be associated with lower empathy, complex emotion recognition, and ToM scores.

## Methods

### Participants

The sample was part of the EU-AIMS LEAP cohort (Charman et al., 2017; Loth et al., 2017). Data were acquired at five sites: Mannheim Central institute of Mental Health (CIMH, Germany), King’s college London (KCL, United Kingdom), University Nijmegen Medical Centre (RUNMC, Netherlands), University Campus BioMedico (UCBM, Italy), and University Medical Centre Utrecht (UMCU, Netherlands). Participants were recruited from a variety of sources including existing volunteer databases, existing research cohorts, clinical referrals from local outpatient centers, special needs schools, mainstream schools and local communities (Charman et al., 2017). The LEAP study was approved by the local ethical committees of the participating centers. Written informed consent was obtained from all participants and/or their legal guardians (for participants under 18 years of age). LEAP participants were included in three age groups [children (6–11 years), adolescents (12–17 years), and adults (18–31 years)], aligning with transitions between formal education (i.e., from primary to secondary school at age 12, and the end of formal education at age 18).

Of all LEAP participants without intellectual disability (N = 653), we selected those who had clean EEG data (for criteria see SI2) with an accompanying MRI head model, as well as parent-reported (children and adolescents) or self-reported (adults) autistic trait scores available, resulting in a sample of 344 individuals. The groups of included and excluded autistic participants differed at the uncorrected group level on SRS-2 raw score and ADI-R social and communication subscales, with higher symptomatology in the excluded group. Of the non-autistic participants, the excluded group had higher SRS-2 raw scores (see SI1 for more details). This might be expected since participants with higher symptom expression are less likely to be able to comply with instructions, and more likely to cause artifacts during EEG and MRI acquisition by moving.

Autistic individuals had an existing clinical diagnosis of ASD according to DSM-IV (American Psychiatric Association, 1994), DSM-IV-TR (American Psychiatric Association, 2000), DSM-5 (American Psychiatric Association, 2013), or ICD-10 criteria (World Health Organization, 2013). Autism characteristics were assessed using the Autism Diagnostic Observation Schedule second edition (ADOS-2; Lord et al., 2012) and the Autism Diagnostic Interview-Revised (ADI-R; Rutter et al., 2003). We did not exclude participants with a clinical autism spectrum diagnosis who did not reach cut-off scores on these instruments or the SRS-2, since clinical judgement has shown to be more stable than diagnostic instrument scores alone (Charman & Gotham, 2013; Lord et al., 2006).

Non-autistic participants (the comparison group) had t-scores below 70 on the Social Responsiveness Scale second edition (SRS-2; Constantino & Gruber, 2012), and had no parent- or self-reported psychiatric conditions. Participants were allowed to take their usual medication at the time of the study.

### Clinical and Cognitive Measurements

All measures were collected as part of a multimodal assessment within LEAP. Cognitive and EEG measures were taken on different assessment days that took place within 4 weeks of one another. Questionnaires were filled out online prior to, during, or shortly after the assessment days. All social cognition measures within LEAP that were stand-alone (e.g., not assessed within neuroimaging environments) were included in the current study.

We used the standard version of the SRS-2 as a dimensional measure of autistic traits. The SRS-2 is a well-validated, 65-item screening instrument that assesses autistic traits over the past six months with subscales on social communication, awareness, motivation, social cognition, and behavior flexibility (Constantino & Gruber, 2012). Each item poses a statement and is scored on a 4-point Likert-scale ranging from ‘not true’ to ‘almost always true’, where higher scores indicate more autistic traits. Parent-report forms were used for the child and adolescent groups, while adults self-reported autistic traits [general internal consistency: 0.94—0.96; Cronbach’s alpha of current study: 0.91 (children), 0.90 (adolescents), 0.93 (adults)]. In the current study, raw scores were used as recommended when considering covariates in research settings.

IQ was assessed using the Wechsler Abbreviated Scales of Intelligence (Wechsler, 1991, 2003) or Wechsler Intelligence Scale for Children (Wechsler, 1997, 2008).

Empathy was measured using the Empathy Quotient (EQ; Wheelwright & Baron-Cohen, 2004). The EQ is a rating scale measuring both cognitive and affective empathy comprising 27 (child version) or 40 (adolescent and adult version) items. Cognitive empathy is the ability to imagine someone else’s mental state, whilst affective empathy is the drive to respond to another person’s mental state with an appropriate emotion. A 4-point Likert scale assesses statements about real life situations, experiences, and interests where empathizing abilities are required. It has been shown to have good test–retest reliability (Auyeung et al., 2012). Cronbach’s alpha internal consistencies in the present sample were 0.90, 0.94, and 0.91, for children, adolescents, and adults respectively. In LEAP, raw scores were binarized so that answers corresponding to higher empathy were scored 1 and those corresponding to lower empathy were scored 0 (Wright & Skagerberg, 2012). Binary scores were then summed into total scores per participant. Thus, higher scores indicate more empathy. EQ forms were filled out by parents for children and adolescents, while adults used self-report forms.

Complex emotion recognition was measured by the Reading the Mind in the Eyes Test (RMET; Baron-Cohen et al., 2001, 2015). The RMET is a well-validated 36-item task with age-appropriate versions for children, adolescents and adults. It requires participants to identify complex emotional expressions from pictures of human eyes and the immediate surrounding facial area by selecting labels that describe emotional states. While the RMET was initially designed to assess ToM ability, growing evidence indicates that it may rather capture complex emotion recognition (Oakley et al., 2016). The RMET has shown good validity across different cultural settings and ages (Doyle-Thomas et al., 2015; Hayward & Homer, 2017; Holt et al., 2014; Lee et al., 2020; Vellante et al., 2013). We used percentage correct scores (the proportion of correctly identified items).

ToM skills were measured by the Frith-Happé Animated Shapes Narratives task (Abell et al., 2000). Participants were asked to verbally describe animations of two triangles moving on a screen in three different conditions: (1) moving in a goal-directed fashion (chasing, fighting), (2) moving interactively with implied intentions (coaxing, tricking), or (3) moving randomly. For this study, we used accuracy scores (how accurately did the participant understand the ToM animation?) on the second condition (ToM). We additionally calculated accuracy scores on the third condition (random movement) as a secondary control measure (how accurately did the participant understand that movement was random or purposeless?). All age groups used the same version as supported by a recent meta-analysis that indicated similar task-effects across autistic children and adults (Wilson, 2021). For further details on all measures used, see (Loth et al., (2017).

### EEG Preprocessing and Calculation of Graph Metrics

Resting-state EEG data were recorded for four minutes (8 blocks of 30 s). Blocks alternated between eyes open and eyes closed conditions, in which participants respectively fixated on a physical hourglass or had their eyes closed. In line with previous resting-state studies, the current study used eyes closed data only, minimizing the impact of blink, eye-movement, or saccade artifacts (e.g., Shou et al., 2017), and facilitating a more focused investigation of neural dynamics, given that eyes-open and eyes-closed conditions may yield distinct results (Petro et al., 2022). In short, the resting-state EEG data were preprocessed, source-localized, and split into the four frequency bands of interest [delta (2–4)Hz, theta (4–8)Hz, alpha (8–13)Hz, and beta (13–30)Hz]. Subsequently, wPLI connectivity matrices were constructed per frequency band, per participant. Connectivity matrices were thresholded and binarized for 10 evenly spaced thresholds between 0.05 (preserving only the top 5 percent of weights) and 0.3 (preserving only the top 30 percent of weights (van Wijk et al., 2010).

Four graph metrics were extracted from each connectivity matrix. Global efficiency describes the number of edges that are needed to reach other nodes in a network (Rubinov & Sporns, 2010) and higher global efficiency indicates more efficient long-range information integration. Characteristic path length is the inverse of global efficiency. While both metrics describe the same network property, global efficiency is better suited to deal with networks containing unconnected nodes (which are likely to occur) and was therefore our preferred metric to describe global network efficiency (Rubinov & Sporns, 2010). We additionally derived path length as it is commonly used in small-world coefficient calculations (described below). We assumed that if path length and global efficiency yielded similar results, path length could be safely used in small-world coefficient calculations. The average clustering coefficient describes how close neighboring nodes in a network cluster together and a higher clustering coefficient indicates increased segregation (or higher efficiency in local information processing; Rubinov & Sporns, 2010). The small-world coefficient reflects the ratio between normalized clustering and path length, and a higher coefficient indicates higher over-all network efficiency (Rubinov & Sporns, 2010). More details on preprocessing, source localization, graph thresholding, and calculation of graph metrics can be found in the supplementary information (SI2).

### Statistical Analyses

To compare demographic, clinical, and social cognition measures between autistic and non-autistic comparison groups within each age group we used Welch two-tailed t-tests, Chi square tests. Note that all comparisons between autistic and comparison groups were conducted per age group given the respective differences in versions and/or informants of social cognition measures across the age groups. This allowed us to account for possible nonlinear age dependencies (e.g., effects only in adolescents but not children or adults), to explore associations between graph metrics and social cognition measures and is in line with previous LEAP studies using predefined age groups (Crawley et al., 2020; Haartsen et al., 2022). Where possible (i.e., measures for which the same version and informant were used across all age groups), measures were compared between age groups for autistic and non-autistic individuals separately.

To compare graph metrics between the autistic and comparison groups we used linear regression models for each graph threshold within each frequency for children, adolescents, and adults separately, where group was the predictor and the graph metric the dependent variable. Analyses were adjusted for site differences, as well as age (squared), sex, and IQ (all fixed effects), as previous studies suggest associations between the latter three and functional connectivity (e.g., Alaerts et al., 2016; Nentwich et al., 2020; Tarokh et al., 2010). Linear predictors were mean centered before entering the statistical models. To account for multiple comparisons across graph metrics, frequency bands, and thresholds, we adopted a cluster-based approach in which group differences for the graph metrics were considered significant only if results (*p* < 0.05) were replicated across at least three subsequent thresholds within the same frequency band (van Wijk et al., 2010). Of these, the threshold showing the highest effect size (determined by partial *R*^*2*^) of all successive significant neighbors was selected for subsequent analyses of the relation between graph metrics and social cognition. Traditional application of false discovery rates or equivalents would be inappropriate here because (1) the successive thresholded networks and thus their resulting graph metrics are not independent for each successive network results from adding a few edges to the previous one, and (2) the small-world coefficient is not independent from clustering and path length, as it reflects the ratio between them. We tested whether any identified autism-related graph metric differed between age groups using ANOVA’s (combined with Tukey’s HSD post-hoc tests).

To investigate associations between the graph metrics and autistic trait scores (SRS-2) across both autistic and non-autistic participants in a dimensional approach, we used linear regression models where SRS-2 scores were the predictor and the graph metric was the dependent variable, including the same covariates. Densities with significant autistic trait associations were determined using the same cluster-based approach as described above. We additionally tested SRS-2 by group interactions within significant densities to check if associations differ for the autistic and non-autistic groups.

To explore associations of the graph metrics (selecting only those that were different between the autistic and comparison groups) with social cognition measures across the total sample of autistic and non-autistic individuals within each age group (as we expected measures to be dimensionally distributed), we used linear regression models including the same covariates as above. We additionally explored interaction effects between graph metrics and SRS-2 scores to see if possible associations depend on autistic traits.

A significance threshold of *p* < 0.05 was applied to all regression models. We present Cohen’s d, η^2^, or partial *R*^*2*^ effect sizes where appropriate. All statistical analyses were performed in R (version 4.0.4; https://www.r-project.org/) and linear regressions were fitted using the lme4 package (Bates et al., 2015). To evaluate the impact of medication on the results, post-hoc sensitivity analyses were performed on significant models by including medication status as a covariate. Significant models were verified to have normally distributed residuals (visual inspection of QQ plots and Shapiro–Wilk normality tests), which did not show autocorrelation (by Durbin-Watson tests) and no homoscedasticity of error variance (visual inspection); there was also no multicollinearity between predictors (determined by variance inflation factors).

## Results

### Demographic and Clinical Descriptives

Table 1 summarizes the main demographics and clinical characteristics of the autistic and comparison groups for each age group. The final sample comprised 184 autistic and 160 non-autistic participants who met our inclusion criteria (for attrition rates see SI3); 86% of 49 autistic children, 97% of 68 autistic adolescents, and 91% of 67 autistic adults scored above the ADOS-2 and/or ADI-R cutoffs, following threshold definitions by Risi et al. (2006), while respectively 14% (7 children), 3% (2 adolescents), and 9% (6 adults) did not reach cut-off thresholds relying on clinical diagnosis alone (for more details and distributions of ADOS-2 scores see SI4).

**Table 1:** Demographic, clinical and social cognitive characteristics of the samples

	Children			Adolescents		Adults	
	Autistic	Comparison		Autistic	Comparison	Autistic	Comparison
n (female)	49 (18)	43 (18)		68 (16)	56 (19)	67 (21)	61 (17)
Age (years)	9.63 (1.39) [6.8–11.9]	9.72 (1.52) [6.9–12.0]		14.8 (1.78) [12.1–17.9]	15.4 (1.72) [12.2–18.0]	23.0 (3.46) [18.0–30.3]	23.1 (3.48) [18.3–31.0]
Full-scale IQ	107 (14.3) [77–139]	112 (13.3) [76–142]		100 (14.9) [75–139]	105 (13.3) [77–126]	106 (13.7) [75.6–148]	109 (11.4) [86.0–142]
Medication	20 (*n* = 47)	0 (*n* = 41)		25 (*n* = 63)	4 (*n* = 54)	15 (*n* = 61)	1 (*n* = 53)
*SRS-2*							
Raw scores	90.6 (31.9) [32–163]	19.0 (13.3) [2–74]		92.0 (28.5) [22–149]	22.1 (16.7) [1–74]	78.5 (30.2) [12–152]	29.5 (14.0) [8–76]
t-scores	73.2 (11.6) [49–90]	44.8 (5.24) [37–66]		73.7 (11.2) [45–90]	46.0 (6.60) [38–66]	63.4 (10.6) [40–89]	46.2 (5.00) [39–63]
*ADOS-2*							
SA CSS	5.29 (2.33) [1–9]	NA		6.42 (2.59) [1–10] (*n* = 67)	NA	5.52 (2.61) [1–10]	NA
RRB CSS	3.91 (3.09) [1–9]	NA		4.49 (2.55) [1–10] (*n* = 67)	NA	4.45 (2.61) [1–10]	NA
total CSS	4.69 (2.40) [1–10]	NA		5.61 (2.74) [1–10] (*n* = 67)	NA	4.78 (2.59) [1–10]	NA
*ADI-R*							
Social	13.9 (7.14) [1–29] (*n* = 47)	NA		17.7 (6.28) [2–29]	NA	13.6 (6.17) [0–26]	NA
Communication	12.3 (5.03) [3–23] (*n* = 47)	NA		14.1 (5.74) [1–26]	NA	10.6 (5.33) [0–24]	NA
RRB	3.91 (3.09) [0–10] (*n* = 47)	NA		4.41 (2.88) [0–12]	NA	4.03 (2.63) [0–12]	NA

Values represent mean (standard deviation) [minimum-maximum]. *SRS-2* Social Responsiveness Scale-2 (note that the SRS-2 t-score was used as a selection criterion only for non-autistic participants [t < 70] and not for autistic participants), *ADOS-2* Autism Diagnostic Observation Schedule-2, *SA* Social Affect, *CSS* Calibrated Severity Scores, *RRB* Restricted and Repetitive Behaviours, *ADI-R* Autism Diagnostic Interview Revised, Medication refers to whether or not participant was using medication affecting the brain at the time of the study (antidepressants, antimigraine, antipsychotics, anxiolytics, hypnotics, sedatives, psychostimulants, analgesics, …)

Age (*p*’s ≥ 0.09), sex (*p*’s ≥ 0.28), and IQ (*p*’s ≥ 0.09) did not show statistically significant differences between autistic and comparison groups within any age group. Autistic trait scores as measured by the SRS-2 were significantly higher in all autistic groups compared to the non-autistic groups (*p*’s ≤ 0.001).

When comparing age groups, full-scale IQ scores were lower for the autistic adolescents compared to the autistic children and adults, who did not significantly differ from each other (F(2,181) = 4.42, *p* = 0.01, η^2^ = 0.05). Full-scale IQ scores in the comparison groups were also lower for the adolescents compared to the children, but not significantly different from the adults. Child and adult comparison groups did not differ on IQ (F(2,157) = 3.83, *p* = 0.024, η^2^ = 0.05). Neither autistic or comparison groups significantly differed between age groups on sex (*p*’s > 0.29) or medication use (*p*’s > 0.17). Autistic age groups did not differ on clinical characteristics measured by the ADOS-2 (*p*’s > 0.38) or ADI-R (*p*’s > 0.29). SRS-2 scores could not be reliably compared between age groups, because of differences in reporters in each age group.

### Social Cognition Descriptives

Table 2 summarizes the social cognitive characteristics of the autistic and comparison groups for each age group. EQ scores were lower for autistic groups compared to the comparison groups for children (t(74.7) = −10.6, *p* < 0.001, d = −2.19), adolescents (t(102.7) = −14.8, *p* < 0.001, Cohen’s d = −2.88), and adults (t(104.7) = −7.9, *p* < 0.001, d = −1.41). RMET scores were lower for autistic groups compared to the comparison groups for adolescents (t(103.8) = −3.0, *p* = 0.003, d = −0.56) and adults (t(116.0) = −2.7, *p* = 0.009, d = −0.47), but not children (t(77.2) = −1.23, *p* = 0.22, d = −0.27). Autistic and comparison groups did not show statistically significant differences in animated shapes ToM or random condition scores for any of the age groups (*p*’s ≥ 0.13).

When comparing age groups, autistic adults scored higher on the animated shapes ToM condition compared to the autistic children and adolescents, who did not significantly differ from each other (F(2,170) = 17.1, *p* < 0.001, η^2^ = 0.17). This pattern was similar for the comparison groups, but the non-autistic children scored significantly lower than the adolescents and adults, while non-autistic adolescent and adult groups did not show statistically significant differences (F(2,139) = 4.76, *p* = 0.01, η^2^ = 0.06). RMET and EQ scores could not be reliably compared between age groups, because of differences in versions and/or reporters in each age group.

**Table 2:** Social cognitive characteristics of the samples

	Children		Adolescents		Adults	
	Autistic	Comparison	Autistic	Comparison	Autistic	Comparison
EQ	13.5 (5.24) [4–27] (*n* = 47)	23.1 (2.94) [14–27] (*n* = 38)	14.7 (6.18) [6–35] (*n* = 56)	31.9 (5.69) [15–39] (*n* = 49)	21.6 (8.33) [4–39] (*n* = 64)	31.5 (4.93) [19–39] (*n* = 54)
RMET	58.2 (13.8) [14–86] (*n* = 42)	61.5 (10.8) [36–82] (*n* = 41)	54.7 (12.9) [26–89] (*n* = 60)	61.0 (8.66) [42–78] (*n* = 51)	65.8 (14.0) [19–89] (*n* = 64)	71.7 (10.4) [44–89] (*n* = 60)
AS ToM	2.35 (1.47) [0–6] (*n* = 48)	2.76 (1.38) [0–6] (*n* = 38)	3.10 (1.64) [0–7] (*n* = 60)	3.60 (1.65) [0–7] (*n* = 47)	4.18 (1.84) [0–8] (*n* = 65)	3.75 (1.67) [0–7] (*n* = 57)
AS random	2.46 (1.17) [0–4] (*n* = 48)	2.63 (1.40) [0–4] (*n* = 38)	2.92 (1.42) [0–4] (*n* = 60)	2.81 (1.26) [0–4] (*n* = 47)	2.71 (1.34) [0–4] (*n* = 65)	2.56 (1.36) [0–4] (*n* = 57)

Values represent mean (standard deviation) [minimum–maximum]. *EQ * Empathy Quotient total scores, *RMET * Reading the Mind in the Eyes Test % correct, *AS ToM* Animated Shapes accuracy scores on ToM condition, *AS random* AS accuracy scores on random condition

### Graph Metric Comparisons Between Autistic and Non-autistic Individuals and Associations with Autistic Trait Scores

Since path length showed similar results as global efficiency for all frequency bands, thresholds, and age groups (SI7), we concluded that it could be reliably used in small-world coefficient calculations. In none of the following analyses did medication status contribute to significant models or results in post-hoc sensitivity analyses (not shown), indicating that taking psychoactive medication had no effect on the present results. Only in the alpha frequency band, graph metrics differed between autistic and comparison groups and were associated with autistic trait scores, but not in the delta, theta, or beta bands.

**Fig. 1:**
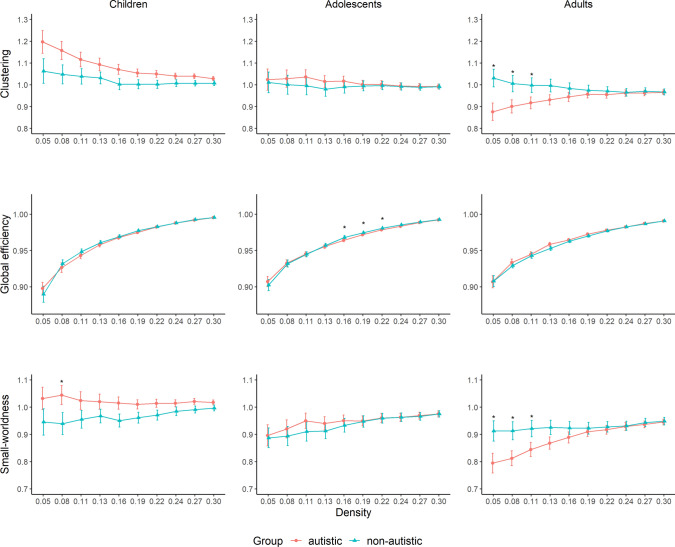
Comparisons of graph metrics between autistic and non-autistic groups in the alpha band. Group means per threshold are shown for each graph metric per age group for the alpha frequency band. Vertical bars represent standard errors. *p < .05

In children, autistic and comparison groups did not significantly differ on any of the graph metrics (Fig. 1) and autistic trait scores were not related to graph metrics in the total sample across the autistic and comparison groups (not shown). Group means and standard deviations for each graph metric, frequency band and threshold for each age group can be found in the supplementary information (SI5), additional data are available upon request.

**Fig. 2:**
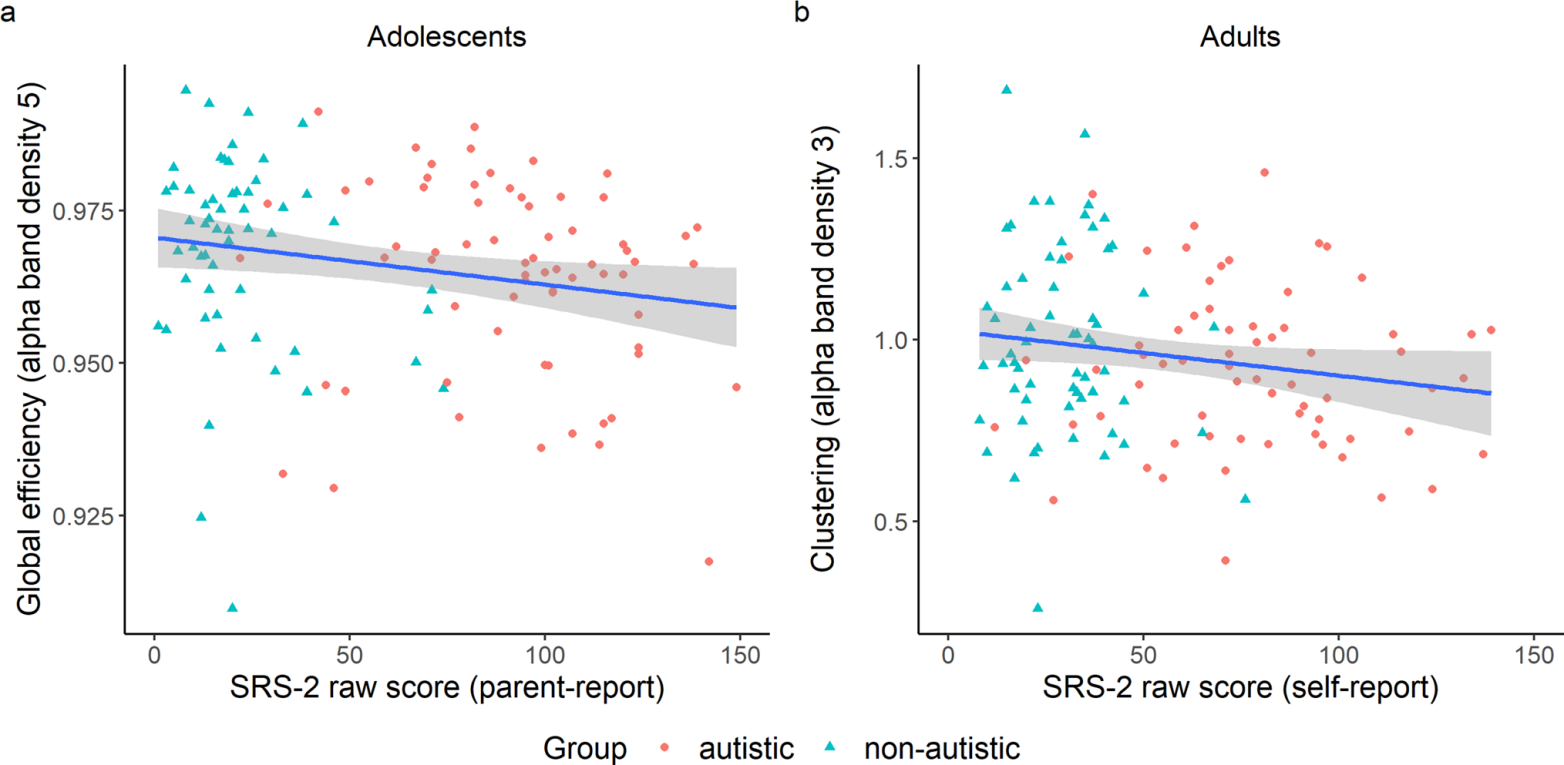
Associations between graph metrics and autistic trait scores across autistic and non-autistic groups. *SRS-2* Social Responsiveness Scale-2. Illustration of association between global efficiency in adolescents and clustering in adults with autistic traits scores for those densities that showed the strongest associations (threshold 0.16 in the alpha band (**a**) and threshold 0.11 in the alpha band (**b**) respectively)

In adolescents, however, global efficiency was lower (*p* < 0.05 at three successive thresholds) for the autistic compared to the comparison group in the alpha band (thresholds 0.16, 0.19, 0.22; Fig. 1 and SI5).We selected threshold 0.22 for subsequent associations with social cognition, based on the largest effect size of group (*R*^2^ = 0.05). Across both the autistic and comparison groups, lower global efficiency was significantly associated with higher autistic trait scores in adolescents for the same thresholds (0.16, 0.19, and 0.22), and additionally for threshold 0.24 (Fig. 2a [visualization of threshold 0.16] and SI6). Effect sizes of autistic trait scores with global efficiency were about twice as high as those for autistic versus comparison group comparisons in global efficiency (*R*^*2*^’s > 0.07). Of the covariates, only site significantly contributed to associations with global efficiency, indicating that participants in London on average had higher global efficiency compared to participants in Mannheim and Nijmegen.

In adults, clustering and small-worldness in the alpha band were significantly lower in the autistic group compared to the comparison group (thresholds 0.05, 0.08, and 0.11; Fig. 1 and SI5). None of the covariates consistently contributed to the associations. We selected threshold 0.05 for clustering and 0.08 for small-worldness for subsequent associations with social cognition measures (*R*^2^ = 0.06 and *R*^2^ = 0.05). Across autistic and comparison groups, less clustering was associated with higher autistic trait scores for thresholds 0.08, 0.11 and 0.13 (Fig. 2b and SI6). Small-worldness did not reach significance on more than two thresholds, and thus did not show evidence of association with autistic trait scores. Subsequently tested autistic trait scores-by-group interactions were not significant for any of the associations with autistic traits in either adolescents or adults, indicating that associations between autistic trait scores and graph metrics were independent of group status (SI6).

Across age groups, autistic groups did not differ in alpha global efficiency. However, non-autistic children on average had higher alpha global efficiency than non-autistic adults, while adolescents did not significantly differ from children or adults in the non-autistic sample (F(2,157) = 4.34, *p* = 0.01, η^2^ = 0.05). Further, autistic children on average had higher clustering and small-worldness than autistic adolescents and adults. Autistic adolescents did not significantly differ from autistic adults on clustering (F(2,181) = 10.9, *p* < 0.001, η^2^ = 0.11), but had lower small-worldness (F(2,181) = 12.43, *p* < 0.001, η^2^ = 0.12). In contrast, comparison age groups did not show statistical differences on clustering or small-worldness, suggesting a stable local efficiency across age.

### Associations Between Autism-Related Graph Metrics and Social Cognition

**Fig. 3:**
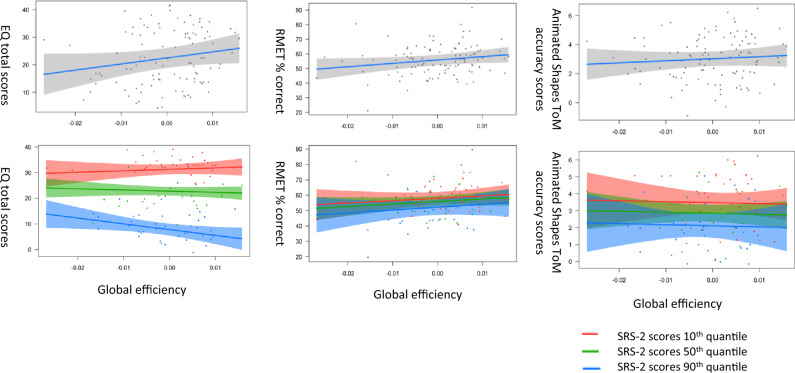
Associations between global efficiency and social cognition measures across autistic and non-autistic adolescents. *SRS-2* Social Responsiveness Scale-2 raw scores. The upper row shows the outcome variables EQ total scores, RMET % correct, and animated shapes ToM scores, respectively, regressed on global efficiency (E) in the alpha band. Global efficiency did not predict EQ or Animated shapes ToM scores, but was associated with RMET % correct with a *p*-value of .053. The second row shows visualizations of the added interaction term (E*SRS-2) for each model. A significant interaction term was found for EQ total scores (beta = −2.77, *p* = .049). All models were adjusted for age, sex, IQ and site

In the total sample of adolescents (autistic and non-autistic individuals together), global efficiency was not predictive of EQ or animated shapes ToM scores, but was tentatively associated with RMET scores [partial R^2^ = 0.04, *p* = 0.053 (Table 3)], suggesting that higher global efficiency is associated with better RMET performance. Age and IQ significantly contributed to this model, indicating that RMET performance increases with age and IQ. The site contrast London-Utrecht was also significant, i.e., participants in London on average scored higher on the RMET compared to participants in Utrecht when adjusting for the other predictors. In the model predicting animated shapes ToM scores, IQ was a significant contributor, i.e., animated shapes ToM performance increased with IQ. The subsequently added interaction between global efficiency and SRS-2 scores significantly predicted EQ scores, but not RMET or animated shapes ToM scores (Table 3). Visualization of this effect shows that in adolescents with high autistic trait scores (above the 90th percentile), lower global efficiency was associated with higher EQ scores, while this association was significantly less strong for individuals with lower autistic trait scores (50th and 10th quantile; Fig. 3).

**Table 3:** Predicting social cognition by graph metrics across autistic and non-autistic adolescents

	EQ (n = 105)			RMET (n = 111)			AS ToM (n = 107)			AS Rand (n = 107)		
	Beta	*p*	*R*^*2*^	Beta	*p*	*R*^*2*^	Beta	*p*	*R*^*2*^	Beta	*p*	*R*^*2*^
Global efficiency	224	.16	.03	237	.053	.04	14.22	.44	.01	−0.12	.23	.01
Sex	−3.83	.10	.03	−3.12	.17	.02	0.19	.57	< .01	−0.31	.28	.01
Age^2^	0.03	.11	.03	0.06	.003*	.08	< 0.01	.42	.01	< 0.01	.73	< .01
IQ	0.09	.30	.01	0.21	.007*	.07	0.03	.007*	.07	< 0.01	.73	< .01
Site	4.71	.17	.03	−8.65	.01*	.08	0.3	.57	.06	0.51	.23	.05
Global efficiency*SRS	−2.77	.049*	.04	0.29	.91	< .01	−0.01	.98	< .01	0.02	.96	< .01

*EQ* Empathy Quotient total score, *RMET* Reading the Mind in the Eyes Test % correct, *AS ToM* Animated Shapes accuracy score on the ToM condition. *AS Rand* Animated Shapes accuracy score on the random condition, *R*^*2*^ partial *R*^*2*^, *global efficiency* global efficiency in the alpha band threshold 0.22, *site* UMCU-KLC contrast. The model predicting EQ was not significant (*R*^*2*^ = .10, *R*^*2*^ adjusted = .04; F(7,97) = 1.54, *p* = .16). With the global efficiency*SRS-2 interaction, the model explained 80% of variance (R^*2*^ adjusted =.78; F(9,95) = 42.5, *p* < .001). The model predicting RMET scores explained 22% of variance (*R*^*2*^ adjusted = .17; F(7, 103) = 4.24, *p* < .001) and with the global efficiency*SRS-2 interaction included it explained 26% of variance (*R*^*2*^ adjusted = .19; F(9,101) = 3.89, *p* < .001). The model predicting animated shapes ToM scores explained 17% of variance (*R*^*2*^ adjusted = .12; F(7,99) = 2.99, *p* < .01), and with the E*SRS interaction included explained 20% of variance (*R*^*2*^ adjusted = .13; F(8,98) = 2.98, *p* < .005). The control model predicting animated shapes random scores did not reach significance with (*R*^*2*^ = .08, *R*^*2*^ adjusted = .02; F(7,99) = 1.24, *p* = .29) or without (*R*^*2*^ = .08; *R*^*2*^ adjusted = −.004; F(9,97) = 0.95, *p* < .48) the interaction

In the total sample of adults, neither alpha clustering nor small-worldness was associated with EQ, RMET, or animated shapes (ToM) scores based on the main or interaction effect models (p’s > 0.20; SI8, SI9). Interestingly, the control model showed that higher small-worldness was significantly associated with lower animated shapes random scores [beta = −1.10, *p* = 0.03, R^2^ = 0.04; the whole model explained 21% of variance (*R*^*2*^_adjusted_ = 0.15; F(8,113) = 3.78; *p* < 0.001)]. The control model predicting animated shapes random scores by global efficiency in adolescents was not significant.

## Discussion

The aim of the current study was to investigate autism-related differences in resting-state EEG functional network organization through graph metrics across development, and to explore their association with domains of social cognition implicated in autism. While effect sizes were modest, we found that autism was associated with less efficient long-range network efficiency in adolescence, and less efficient local and overall network connectivity in adulthood as compared to non-autistic individuals, supported by dimensional analyses in the total sample. We found no significant differences (i.e., failed to reject the null-hypothesis) between autistic and non-autistic children on these measures; however, larger samples with predefined equivalence margins are required to test whether or not there are indeed no meaningful differences in childhood. Moreover, our findings suggest that long-range network efficiency may play a role in empathic behaviors and complex emotion recognition as assessed by the RMET during adolescence, which is a critical phase for the development of social cognition.

### Network Topology

As expected, we found different patterns of autism-related network topology across different age groups. Autistic adolescents demonstrated less efficient long-range network efficiency (global efficiency) compared to non-autistic adolescents. In contrast, autistic adults exhibited less efficient local connectivity (clustering) and had networks that less closely resembled optimal small-world topologies compared to non-autistic adults. These findings were largely supported by dimensional analyses across the autistic and comparison groups together: less efficient long-range network connectivity in adolescents and reduced local connectivity in adults were associated with higher levels of autistic traits. All observed differences in network topology in adolescents and adults were confined to the alpha frequency range (8–13 Hz), which resonates with studies that have indicated the importance of alpha activity in brain maturation (Miskovic et al., 2015), large-scale integration of cortical information (Palva & Palva, 2011), and early development in autism (Orekhova et al., 2014).

Our findings of autism-related differences in resting-state EEG network-topology suggest that graph metrics could provide valuable information to identify differences in functional connectivity in autism in addition to overall resting-state functional connectivity, as a recent evaluation of wPLI resting-state EEG data from the LEAP cohort did not show differences between autistic and non-autistic individuals (Garcés et al., 2022). Our results extend findings by the few previous EEG resting-state studies that also used graph metrics. In adults, Barttfeld et al. (2011) also observed lower local connectivity (clustering) in autistic compared to non-autistic individuals, but their study was restricted to the delta band. They additionally found higher path length (less efficient long-range connectivity) in autistic adults in the delta band, whereas in the current study less efficient long-range-connectivity was confined to the adolescent sample in the alpha band. However, as the Barttfeld sample adopted a wide age range including adolescents (16–38 years), their observation of less efficient long-range connectivity may have been driven by the younger participants in their sample. Contrary to previous findings of reduced path length and clustering in the alpha band in autistic children (7–13 years; Zeng et al., 2017), we found no atypical network topologies in autistic children. Methodological differences between the work of Zeng et al. (2017) and the current study may have contributed to this discrepancy, including differences in sample size (42 vs 92 children), functional connectivity measure (PLI vs. wPLI), type of resting-state data (eyes open vs eyes-closed), and reference (mastoid vs. average).

Cross-sectional analyses may suggest developmental changes across age groups in the identified autism-related network metrics. Long-range connectivity decreased from childhood to adulthood in non-autistic but not in autistic participants. This indicates that the resting-state network has a more integrated structure in childhood compared to adulthood in typical development, but that network integration remains relatively stable over this period in autism. While this seems contradictory to a recent review of fMRI studies suggesting that in typical development network integration increases until about 40 years (Edde et al., 2021), our results do corroborate previous EEG resting-state findings that path length (inverse global efficiency) in the alpha band increases throughout adolescence and young adulthood in typical development (Smit et al., 2010). This suggests that long-range network efficiency follows a different developmental trajectory in autism, with the strongest difference in adolescence (i.e., less long-range connectivity in autistic adolescents). Local connectivity and small-worldness both decreased from childhood to adulthood in autistic individuals, while these network properties were stable across age in non-autistic individuals. This again is consistent with previous EEG findings in relation to typical development (Smit et al., 2010), and suggests that autism is associated with a decrease of local connectivity and overall network efficiency across development into adulthood, with the strongest difference in adulthood (i.e., less local connectivity in autistic adults), suggesting premature decline. The lack of significant network differences between autistic and non-autistic children in the current study could imply that variations in brain networks associated with autism might arise as a result of more profound environmental interactions in autistic individuals later in development. Possibly, the increasing social demands experienced during adolescence (Blakemore, 2012; Blakemore & Mills, 2014; Kilford et al., 2016) could be perceived differently by autistic individuals, leading to distinct effects on the reorganization of brain networks during this developmental phase (Scherf et al., 2012) or become more prominent during adulthood (as seen in local and overall network efficiency), as compared to their non-autistic peers. However, this interpretation remains speculative, as the absence of significant findings does not confirm the absence of true differences.

### Associations Between Network Topology and Social Cognition

As expected, autistic individuals in all age groups showed less empathic behaviors in daily life (EQ) and lower complex emotion recognition performance (RMET) compared to non-autistic individuals, except for autistic children, who did not differ from non-autistic children on complex emotion recognition performance. Surprisingly, autistic and non-autistic individuals did not differ on implicit ToM ability (animated shapes task) in any of the age groups. Previous studies have reported reliable differences in performance between autistic and non-autistic individuals on both the RMET and animated shapes task across all ages (Wilson, 2021). A possible reason for our unsuccessful replication may be that our large sample better captured the heterogeneity in cognitive features associated with autism compared to the smaller samples used in earlier studies, which may have tapped into specific subsamples within the autism population (Lombardo et al., 2019). Independent of autism diagnosis, adults scored higher on implicit ToM ability than children. For autistic individuals there seems to be a developmental delay: performance of autistic adolescents was similar to autistic children and did not yet reach adult levels, while non-autistic adolescents already performed at adult level. However, this delay did not result in significant differences between autistic and non-autistic adolescents.

We found some support for the hypothesis that autism-related network topologies may be associated with social cognition in adolescents, however not in adults. While associations were weaker and more complex than expected, results show preliminary support for the underconnectivity hypothesis of autism during adolescence. It states that underconnectivity in integrative neural networks, that largely depend on long-range connectivity, may affect higher-order cognitive functions including social cognition (Just et al., 2004). In line with this, adolescents with less efficient long-range network connectivity tended to have lower complex emotion recognition ability. While being at near significant level, it should be considered that the effect size was in the small-to-moderate range. EEG activity during task engagement may have shown stronger associations than resting-state EEG, which should be explored in future studies. There were, however, no indications for associations between long-range connectivity and ToM ability as measured by the animated shapes task. This is in line with the lack of statistically significant group differences in task performance between autistic and non-autistic individuals. Interestingly, in adolescents with high autistic trait scores, higher efficiency of long-range network connectivity was related to fewer rather than more parent-reported empathic behaviors in daily life. This may suggest that adolescents with high autistic trait scores and relatively high global efficiency utilize their cognitive efficiency for complex skills other than empathy, at the cost of their capacity to empathize. This hypothesis would be in line with the empathizing-systemizing hypothesis of autism, which proposes that below average empathy in autistic individuals coincides with a relative strength in systemizing (i.e., the drive to analyze or construct systems; Baron-Cohen, 2002, 2009).

In adults, autism-related network topology was not associated with social cognition measures. Having found that only local but not long-range connectivity was different in autistic compared to non-autistic adults, we speculate that, in line with the underconnectivity hypothesis, long-range connectivity plays a bigger role in social cognition than local connectivity. Interestingly, we also found an association in adults between increased small-worldness and less accurate descriptions of videos of the animated shapes random condition; less accurate meaning the attribution of mental states to randomly moving objects that did not have any interaction. It could therefore be reasoned that individuals with stronger small-world topologies (which was also related to lower autistic trait scores), may have been more eager to attribute mental states to randomly moving objects.

### Strengths and Limitations

A strength of this study is the use of data from one of the largest multicenter initiatives aimed at identifying biomarkers in autism across children, adolescents, and adults. This large sample spanning multiple age groups, combined with the sophisticated analyses of graph metrics across multiple frequency bands in relation to social cognition measures, offers a novel contribution to the literature.

Despite the study sample being relatively large for EEG research, statistical power may still have been insufficient to detect smaller brain-behavior correlations, sex differences, or to effectively control for medication use, pubertal staging and co-occurring neurodevelopmental and psychiatric conditions. Regarding sample characteristics, autistic participants who were included in the analysis had lower levels of autistic traits (lower SRS-2 raw scores and ADI-R social and communication subscale scores) compared to those excluded. Consequently, our findings may not generalize to individuals with higher levels of autistic symptomatology. Similarly, the ongoing trend of increased ASD diagnoses, bearing the risk of diagnostic dilution (Russell et al., 2022), may have contributed to the lack of group differences between autistic and non-autistic children.

With respect to our measures, we used different versions and/or informants for social and clinical measurements across age groups, complicating assessment of brain-behavior relationships over the entire age range. Furthermore, while the RMET has been widely used to study both ToM ability and complex emotion recognition, it has also been suggested to capture verbal ability (Peñuelas-Calvo et al., 2019).

Methodological choices regarding the graph measures [e.g., EEG vs magnetoencephalography (MEG), eyes-open vs eyes-closed analyses, binarized vs weighted analyses of connectivity graphs, use of different connectivity metrics, the evaluation of the gamma frequency band] may partly explain discrepancies with prior studies, such as reports of greater alpha-band network efficiency in autism (Kitzbichler et al., 2015). Finally, the translation of graph-theoretic measures of efficiency in the alpha frequency band to computational efficiency in the brain is not straightforward. Although previous studies have demonstrated associations between more efficient EEG-derived networks and cognitive abilities such as IQ (Zakharov et al., 2020), it is important to recognize that graph-theoretic efficiency may not fully account for computational efficiency in the brain.

### Conclusions and Future Research

This cross-sectional study suggests subtle atypicalities in functional network development in autism, and a possible contribution of autism-related network topology to social cognition abilities during adolescence. While the latter present novel findings, future research, preferably in larger samples, is needed to replicate these results. Longitudinal analyses will be crucial to elucidate differences in network topology and connectivity patterns across development. Moreover, studying network topologies during active social-cognitive tasks could offer a more dynamic understanding of neural efficiency in social contexts. In terms of outcome measures, future endeavors should incorporate a broader range of social cognition measures to differentiate between ToM, emotion recognition, and verbal ability. Measuring reaction times may also provide a more sensitive index of individual differences in ToM processing (Paul et al., 2021). Therefore, systematic investigation into the impact of different graph theoretical methodological options and social cognition measures could further advance the field and clarify the robustness of current findings, while determining the specificity to autism in comparison to individuals with diverse neurodevelopmental conditions.

## Supplementary Information

Below is the link to the electronic supplementary material.

Supplementary file1 (DOCX 1363 kb)
