# Supplementary material for: Atypical Resting-State EEG Graph Metrics of Network Efficiency Across Development in Autism and Their Association with Social Cognition: Results from the LEAP Study
Source: J Autism Dev Disord. 2025 Feb 14;56(7):2557–73. doi: 10.1007/s10803-025-06731-0 (PMC13346277; doi:10.1007/s10803-025-06731-0)
Supplement: Supplementary file 1 — Supplementary file1 (DOCX 1363 kb) [file 10803_2025_6731_MOESM1_ESM.docx]

# Supplementary information (SI)

# Atypical resting-state EEG graph metrics of network efficiency across development in autism and their association with social cognition: Results from the LEAP study

*SI1 Representativeness of the LEAP subsample*

|  | Excluded  Autistic  (n = 193) | Excluded  non-autistic  (n = 116) | p-value  autistic group | p-value  non-autistic group |
| --- | --- | --- | --- | --- |
| Sex (male/female) | 152/41 | 77/39 | .06 | 1.0 |
| Age (years) | 16.4 (6.08) | 17.1 (6.17) | .92 | .75 |
| IQ | 102 (15.9) (n=186) | 109 (13.1) (n=115) | .09 | .44 |
| Medication | 30.1% (n=178) | 6.90% (n=106) | .27 | .42 |
| *SRS-2* | | | | |
| Raw score | 91.9 (28.4) (n=149) | 30.0 (21.6) (n=67) | .04 | .03 |
| t-score | 72.1 (11.3) (n=149) | 46.3 (7.08) (n=67) | .20 | .41 |
| *ADOS-2* | | | | |
| SA CSS | 6.19 (2.67) (n=183) |  | .16 |  |
| RRB CCS | 4.99 (2.80) (n=183) |  | .07 |  |
| total CCS | 5.62 (2.88) (n=183) |  | .05 |  |
| *ADI-R* | | | | |
| Social | 17.2 (6.68) (n = 176) |  | <.01 |  |
| Communication | 14.0 (5.63) (n = 176) |  | .01 |  |
| RRB | 4.62 (2.52) (n = 176) |  | .08 |  |

Note: Overview of clinical and demographic characteristics of the excluded participants. Information for the equivalent information on included participants can be found in the main manuscript (Table 1). Also refer to the main manuscript for more information on abbreviations and nomenclature. P-values represent the result of the statistical test (t-test or Chi-square) comparing the included and excluded participants for autistic and non-autistic participants separately.

## **SI2** Additional information on EEG data analyses

### Preprocessing

EEG data were recoded using the following systems: Brainvision (CIMH, KCL, RUNMC), BioSemi (UMCU), and Micromed (UCBM), with sampling frequencies of 5000Hz (KCL, RUNMC), 2048Hz (UMCU), 2000Hz (CIMH), and 256-1000 Hz (UCBM). All sites used caps compliant with the 10-20 system, with 60 to 70 electrodes.

Preprocessing of the LEAP EEG resting state data was conducted using MATLAB (MATLAB Release 2020b, MathWorks Inc., Natick, MA, USA) and the FieldTrip toolbox (Oostenveld et al., 2011). Raw EEG data were resampled to 1000Hz and bandpass filtered to [1-32]Hz. Data from 61 electrodes common to most recording sites were used for subsequent analyses. Channels were re-referenced to FCz. Pre-processing was done manually, starting with elimination of defective or highly noisy channels. Next, sections with large transient muscle or movement artifacts were located and discarded. Independent component analysis (ICA; algorithm: fastICA (<http://www.cis.hut.fi/projects/ica/fastica/>, (Hyvärinen & Oja, 2000)) was performed and independent components (ICs) reflecting ocular, muscular, cardiac, or other artifacts were removed from the data. Data were then checked again and if data still showed signs of any noisy components, preprocessing steps were repeated, starting again from the raw data. Finally, eliminated channels were interpolated and data were re-referenced using the average of all electrodes. Pre-processing was performed blind to the subject’s clinical information and diagnosis. All participants selected for the current study had sufficient clean EEG data available, according to the following criteria: (1) at least 15 clean 2.5-second epochs of eyes-closed data, (2) more than 50 usable channels and fewer than four interpolated neighboring channels, and (3) number of good channels minus number of artefactual independent components over 34 (Garcés et al., 2022). EEG quality metrics per age group are described below and compared between the autistic and comparison groups.

| Children | Autistic group  (n = 49) |  | Comparison group  (n = 43) |  | t-test  p-value |  | Cohen’s d |
| --- | --- | --- | --- | --- | --- | --- | --- |
| Number of interpolated channels | 3.31 (1.78) |  | 2.84 (1.81) |  | .22 |  | 0.26 |
| Number of rejected ICs | 11.90 (4.74) |  | 10.67 (3.83) |  | .17 |  | 0.28 |
| Number of clean trials | 18.45 (4.11) |  | 18.63 (5.15) |  | .86 |  | 0.04 |
| Adolescents | Autistic group  (n = 68) |  | Comparison group  (n = 56) |  | t-test  p-value |  | Cohen’s d |
| Number of interpolated channels | 2.71 (1.59) |  | 2.46 (1.66) |  | .41 |  | 0.15 |
| Number of rejected ICs | 10.47 (4.01) |  | 10.57 (4.27) |  | .89 |  | 0.02 |
| Number of clean trials | 21.31 (4.67) |  | 22.59 (3.64) |  | .09 |  | 0.30 |
| Adults | Autistic group  (n = 67) |  | Comparison group  (n = 61) |  | t-test  p-value |  | Cohen’s d |
| Number of interpolated channels | 2.94 (1.74) |  | 2.72 (1.86) |  | .49 |  | 0.12 |
| Number of rejected ICs | 10.75 (4.35) |  | 9.15 (3.82) |  | .029 |  | 0.39 |
| Number of clean trials | 22.43 (3.98) |  | 22.16 (4.28) |  | .71 |  | 0.07 |

On average, more ICs were removed from the data for autistic adults compared to adults in the comparison group. There were no significant differences between the autistic and comparison groups for the other quality control measures for children, adolescents, or adults.

From a developemental perspective, the number of interpolated channels and the number of rejected ICs did not significantly differ between autistic children, adolescents, and adults (p’s > .12), or between non-autistic children, adolescents, and adults (p’s > .12). Autistic children had fewer clean trials compared to autistic adolescents and autistic adults (p’s < .01), and this pattern was the same for non-autistic participants: non-autistic children had fewer clean trials compared to non-autistic adolescents and non-autistic adults (p’s < .001).

Finally, none of the quality control metrics were significantly associated with autistic trait scores (SRS-2 scores) in children (p’s > 0.27). In adolescents, autistic trait scores were negatively associated with number of clean trials (p < .01), but not with number of interpolated channels (p = .98) or number of rejected ICs (p = 0.87). In adults, autistic traits were positively associated with number of rejected trials (p < .01), but not with number of interpolated channels (p > .54), or number of clean trials (p > .68).

For completeness, we additionally report quality control metrics per site (see SI11).

### Source reconstruction

Pre-processed data were brought into source space using FieldTrip. For this, head models were built from each individual’s T1-weighted MRI scans acquired for LEAP. MRI acquisition is described in detail elsewhere (Holiga et al., 2019). MRIs were segmented into gray matter, white matter, cerebrospinal fluid, bone, soft tissue, and air using SPM12 (Penny et al., 2007). These probabilistic images were then smoothed, thresholded, and resliced to binary masks for brain (gray and white matter and cerebrospinal fluid), skull and scalp tissues separately, and subsequently transformed in FieldTrip to hexahedral meshes. All three tissue types were assumed to have homogeneous and isotropic conductivity, and an age-dependent skull conductivity in line with the BESA (BESA, Gräfelfing, Germany) recommended conductivity ratios. Segmentations were visually inspected, and the forward model was derived using Fieldtrip and SimBio (SimBio development group 2018). Following a 3D cubic diamond grid with 1 cm spacing, 1199 source locations of interest were defined in gray matter in MNI space. Source and electrode positions were transformed from MNI to a subject’s individual space, using a non-linear transformation in SPM12 and source timeseries were estimated with linearly constrained minimum variance beamformer, using a regularization of 5% of the average trace of the covariance matrix, a common filter for resting state eyes open and eyes closed and projecting the source time series into the direction of maximal power (van Veen et al., 1997).

Sources were then grouped into Regions of Interest (ROIs), based on the Automated Anatomical Labelling atlas in MNI space. Subcortical regions were not included in this analysis, so that 80 cortical ROIs remained (40 symmetrical ROIs per hemisphere). The first principal component of all source time series encompassed in a region was taken to be the representative time series of the region.

### Connectivity – weighted Phase Lag Index (wPLI)

Functional connectivity was calculated using FieldTrip. Four second non-overlapping epochs were extracted from eyes-closed sections of the ROI time series. Subsequently, data were split into four commonly used frequency bands of interest (delta [2-4]Hz, theta [4-8]Hz, alpha [8-13]Hz, and beta [13-30]Hz) using Multi-taper Fast Fourier Transform. Spectral smoothing from the mean to the edge of the frequency band was applied. Connectivity values were calculated for each pair of ROIs per frequency band per participant. To estimate functional connectivity, the weighted Phase Lag Index was used. This is a phase synchronization measure that deals with volume conduction by discarding zero- and π-lag synchronization (Vinck et al., 2011).

### Network analysis – graph theory

Graph network analyses were conducted using the MATLAB Brain Connectivity Toolbox (BCT; (Rubinov & Sporns, 2010). Graph theory is a mathematical framework to characterize complex networks as graphs, by representing a network as a system of *n* points (also called nodes) that are connected by *k* links (also called edges). Network metrics can be derived from a graph to describe the topological properties of the network, giving insight into segregation, integration and efficiency of information transport between nodes (Bullmore & Sporns, 2009).

Whole-brain graphs were built using the 80x80 connectivity matrices per frequency band per subject, with ROIs as nodes and wPLI indexes as the edges’ weights. For this, connectivity matrices were thresholded and binarized (van Wijk et al., 2010). Since there is no consensus about the level of thresholding to date and to ensure results were not driven by an arbitrary definition of one threshold, we here opted for a range of ten evenly spaced thresholds between 0.05 (preserving only the top 5 percent of weights) and 0.3 (preserving only the top 30 percent of weights). This resulted in ten binary matrices per frequency band and subject.

Four graph metrics were extracted in total. The average clustering coefficient (C) is defined as the fraction of neighbors of a node that are also each other’s neighbors (i.e., the proportion of connections among neighbors, divided by the maximal number of connections that could exist between the neighbors (Rubinov & Sporns, 2010). The coefficient thus indicates how close neighbors cluster together. A higher clustering coefficient indicates increased segregation (or a higher degree of connectedness in local specialized regions) and thus higher efficiency in local information processing.

Global efficiency is the average inverse shortest path length (characteristic path length) of a network (Rubinov & Sporns, 2010). A short path length indicates that each node in a network can on average reach other nodes through relatively few edges, and thus indicates higher global efficiency of signal propagation. While both global efficiency and path length describe the same network property, the former is better suited to deal with networks containing unconnected nodes (which are likely to occur) and was therefore our preferred choice to measure global integration (Rubinov & Sporns, 2010). We additionally derived path length, as this metric is commonly used in small-world coefficient calculations (described below). We assumed that if path length and global efficiency yielded similar results, path length could be safely used in small-world coefficient calculations.

The small-world coefficient was derived by calculating the ratio between normalized clustering and path length, indicating the degree to which a network is both globally and locally efficient. A higher clustering coefficient indicates a more efficient network. All metrics were normalized by dividing them by values obtained from randomly generated networks (Crand, Lrand, and Erand) of the same size and with the same proportion of connections in a network compared to the total possible connections; C/Crand = Cnorm, L/Lrand = Lnorm, and E/Erand = Enorm ;de Haan et al., 2009).

## **SI3** Attrition rates for participant inclusion

Note: Social Responsiveness Scale (SRS-2) parent-report for children and adolescents, self-report for adults. Non-ID = participants without intellectual disability.

## **SI4** Overview of the of number participants reaching autism thresholds on the ADOS-2and/or ADI-R and distribution of ADOS-2 scores in the autism group

| Scored above threshold on: | ASD children (n=49) | ASD adolescents (n=68) | ASD adults (n=67) |
| --- | --- | --- | --- |
| ADOS-2 | 59% (n=29) | 74% (n=50) | 61% (n=41) |
| ADI-R | 71% (n=35) | 91% (n=62) | 78% (n=52) |
| ADOS-2 + ADI-R | 45% (n=22) | 68% (n=46) | 48% (n=32) |
| Neither ADOS-2 nor ADI-R | 14% (n=7) | 3% (n=2) | 9% (n=6) |
| ADOS-2 total CSS: | | | |
| 1-2 (minimum level of absence of autistic symptoms) | 20% (n=10) | 19% (n=13) | 30% (n=20) |
| 3-4 (Low level of autistic symptoms) | 29% (n=14) | 18% (n=12) | 21% (n=14) |
| 5-7 (Mild level of autistic symptoms) | 37% (n=18) | 34% (n=23) | 34% (n=23) |
| 8-10 (high level of autistic symptoms) | 14% (n=7) | 28% (n=19) | 15% (n=10) |

Note: ADOS-2 = Autism Diagnostic Observation Schedule (second edition), ADI-R = Autism Diagnostic Interview revised. Total CSS = Total Calibrated Severity Scores. Threshold definitions following (Risi et al., 2006). For 2 children ADI-R scores were missing. For one adolescent ADOS-2 scores were missing.

## **SI5** Comparisons of graph metrics between autistic and comparison groups for densities that showed differences between these groups

| *Adolescents* | | | | | | | | | | | |
| --- | --- | --- | --- | --- | --- | --- | --- | --- | --- | --- | --- |
|  | Global Efficiency  (threshold 0.16) | | |  | Global Efficiency  (threshold 0.19) | | |  | Global Efficiency  (threshold 0.22) | | |
|  | Beta | *p* | *R^2^* |  | Beta | *p* | *R^2^* |  | Beta | *p* | *R^2^* |
| Group | 6.59e-03 | .03* | .04 |  | 4.81e-03 | .02* | .05 |  | 3.74e-03 | .02* | .05 |
| Sex | -3.75e-03 | .24 | .01 |  | 2.59e-03 | .24 | .01 |  | 1.65e-03 | .34 | <.01 |
| Age^2^ | -3.34e-05 | .22 | .01 |  | -2.08e-05 | .27 | .01 |  | -2.44e-05 | .09 | .02 |
| IQ | 5.22e-05 | .61 | <.01 |  | -1.91e-05 | .79 | <.01 |  | 9.93e-07 | .99 | <.01 |
| Site | -9.22e-03 | .01* | .07 |  | -6.64e-03 | .003* | .09 |  | -4.42e-03 | .008* | .07 |
| *Adults* | | | | | | | | | | | |
|  | Clustering  (threshold 0.05) | | |  | Clustering  (threshold 0.08) | | |  | Clustering  (threshold 0.11) | | |
|  | Beta | *p* | *R^2^* |  | Beta | *p* | *R^2^* |  | Beta | *p* | *R^2^* |
| Group | 1.5e-01 | .006* | .06 |  | 1.08e-01 | .02* | .04 |  | 9.35e-02 | .045* | .03 |
| Sex | -7.9e-02 | .20 | .01 |  | -5.51e-02 | .29 | .01 |  | -3.89e-02 | .39 | .01 |
| Age^2^ | -1.28e-04 | .48 | <.01 |  | -2.61e-04 | .09 | .02 |  | -2.87e-04 | .03* | .04 |
| IQ | 3.90e-03 | .08 | .02 |  | 1.95e-03 | .30 | .01 |  | 2.19e-03 | .18 | .01 |
| Site | -8.39e-02 | .29 | .07 |  | -4.34e-02 | .51 | .04 |  | -3.58e-02 | .54 | .06 |
| *Adults* |  | | |  |  | | |  |  | | |
|  | Small-worldness  (threshold 0.05) | | |  | Small-worldness  (threshold 0.08) | | |  | Small-worldness  (threshold 0.11) | | |
|  | Beta | *p* | *R^2^* |  | Beta | *p* | *R^2^* |  | Beta | *p* | *R^2^* |
| Group | 1.23e-01 | .018* | .05 |  | 1.06e-01 | .013* | .05 |  | 8.01e-02 | .04* | .04 |
| Sex | -8.59e-02 | .13 | .02 |  | -6.56e-02 | .16 | .02 |  | -4.10e-02 | .33 | <.01 |
| Age^2^ | -1.08e-04 | .51 | <.01 |  | -1.77e-04 | .19 | .01 |  | -2.25e-04 | .07 | .03 |
| IQ | 2.95e-03 | .15 | .02 |  | 1.25e-03 | .45 | <.01 |  | 1.66e-03 | .28 | .01 |
| Site | -9.37e-02 | .19 | .07 |  | -4.75e-02 | .42 | .05 |  | -5.25e-02 | .33 | .07 |

Group = autistic vs comparison, *R^2^* = partial *R^2^*, site = UMCU-KLC contrast. For global efficiency in adolescents, the model for threshold 0.16 explained 12% of variance (*R^2^*_adjusted_=0.06; F(7, 116)=2.22, *p*=.04), the model for threshold 0.19 explained 13% of variance (*R^2^*_adjusted_=.08; F(7, 116)=2.51, *p*=.02), and the model for threshold 0.22 explained 12% of variance (*R^2^*_adjusted_=.06; F(7, 116)=2.29, *p*=.03) in the alpha band. For clustering in adults, the model for threshold 0.05 explained 16% of variance (*R^2^*_adjusted_=0.11; F(8,119)=2.93, *p*=.005), the model for threshold 0.08 explained 14% of variance (*R^2^*_adjusted_=.08; F(8, 119)=2.34, *p*=.02), and the model for threshold 0.11 explained 17% of variance (*R^2^*_adjusted_=.11; F(8, 119)=2.95, *p*=.004) in the alpha band. For small-worldness in adults, the model for threshold 0.05 explained 14% of variance (*R^2^*_adjusted_=0.08; F(8, 119)=2.46, *p*=.01), the model for threshold 0.08 explained 15% of variance (*R^2^*_adjusted_=.09; F(8, 119)=2.56, *p*=.01), and the model for threshold 0.11 explained 17% of variance (*R^2^*_adjusted_=.11; F(8, 119)=3.10, *p*=.03) in the alpha band. For children, none of the metrics showed significant differences between autistic and comparison groups. **p*<.05

## **SI6** Associations between graph metrics and autistic trait scores across autistic and comparison groups

| *Adolescents* | | | | | | | | | | | | | | | |
| --- | --- | --- | --- | --- | --- | --- | --- | --- | --- | --- | --- | --- | --- | --- | --- |
|  | Global Efficiency  (threshold 0.16) | | |  | Global Efficiency  (threshold 0.19) | | |  | Global Efficiency  (threshold 0.22) | | |  | Global Efficiency  (threshold 0.24) | | |
|  | Beta | *p* | *R^2^* |  | Beta | *p* | *R^2^* |  | Beta | *p* | *R^2^* |  | Beta | *p* | *R^2^* |
| SRS | -1.15e-04 | .001^*^ | .09 |  | -7.86e-05 | .002^*^ | .08 |  | -5.71e-05 | .003^*^ | .07 |  | -4.28e-05 | .02^*^ | .07 |
| Sex | 4.06e-03 | .19 | .01 |  | 2.75e-03 | .21 | .01 |  | 1.73e-03 | .31 | <.01 |  | 3.88e-04 | .80 | <.01 |
| Age^2^ | -3.91e-05 | .14 | .02 |  | -2.44e-05 | .19 | .02 |  | -2.67e-05 | .06 | .03 |  | -1.54e-05 | .24 | .01 |
| IQ | 5.55e-06 | .96 | <.01 |  | -4.92e-05 | .49 | <.01 |  | -1.94e-05 | .73 | <.01 |  | -9.82e-06 | .85 | <.01 |
| Site | -1.07e-02 | .02^*^ | .09 |  | -7.56e-03 | .02^*^ | .11 |  | -5.06e-03 | .05 | .08 |  | -5.88e-03 | .01^*^ | .08 |
| SRS*group | -5.63e-05 | .70 | <.01 |  | -6.46e-05 | .53 | <.01 |  | -2.00e-05 | .80 | <.01 |  | -5.49e-06 | .94 | <.01 |
| *Adults* | | | | | | | | | | | | | | | |
|  | Clustering  (threshold 0.05) | | |  | Clustering  (threshold 0.08) | | |  | Clustering  (threshold 0.11) | | |  | Clustering  (threshold 0.13) | | |
|  | Beta | *p* | *R^2^* |  | Beta | *p* | *R^2^* |  | Beta | *p* | *R^2^* |  | Beta | *p* | *R^2^* |
| SRS | -1.66e-03 | .056 | .03 |  | -1.43e-03 | .046* | .03 |  | -1.42e-03 | .02* | .04 |  | -1.20e-03 | .046* | .03 |
| Sex | -8.75e-02 | .17 | .02 |  | -6.35e-02 | .23 | .01 |  | -4.83e-02 | .29 | <.01 |  | -4.78e-02 | .23 | .01 |
| Age^2^ | -1.11e-04 | .55 | <.01 |  | -2.48e-04 | .10 | .02 |  | -2.76e-04 | .04* | .04 |  | -2.32e-04 | .047* | .03 |
| IQ | 3.90e-03 | .09 | .02 |  | 1.84e-03 | .33 | <.01 |  | 1.99e-03 | .23 | .01 |  | 2.21e-03 | .13 | .02 |
| Site | -1.19e-01 | .15 | .07 |  | -7.30e-02 | .28 | .04 |  | -6.47e-02 | .27 | .06 |  | -4.53-e02 | .38 | .04 |
| SRS*group | 1.82e-03 | .60 | <.01 |  | -1.67e-03 | .56 | <.01 |  | -7.84e-04 | .75 | <.01 |  | 8.11e-04 | .71 | <.01 |

SRS-2 = Social Responsiveness Scale-2, *R^2^* = partial *R^2^*, site = UMCU-KLC contrast, group = autistic vs comparison. For global efficiency in adolescents, the model for threshold 0.16 explained 16% of variance (*R^2^*_adjusted_=0.11; F(7, 116)=3.13, *p*=.005), the model for threshold 0.19 explained 17% of variance (*R^2^*_adjusted_=0.11; F(7, 116)=3.28, *p*=.003), the model for threshold 0.22 explained 15% of variance (*R^2^*_adjusted_=0.09; F(7, 116)=2.84, *p*=.009), and the model for threshold 0.24 explained 12% of variance (*R^2^*_adjusted_=0.06; F(7, 116)=2.21, *p*=.039) in the alpha band. For clustering in adults, the model for threshold 0.05 explained 14% of variance (*R^2^*_adjusted_=0.08; F(8, 119)=2.35, *p*=.02), the model for threshold 0.08 explained 13% of variance (*R^2^*_adjusted_=0.07; F(8, 119)=2.17, *p*=.03), the model for threshold 0.11 explained 17% of variance (*R^2^*_adjusted_=0.12; F(8, 119)=3.13, *p*=.003), and the model for threshold 0.13 explained 15% of variance (*R^2^*_adjusted_=0.10; F(8, 119)=2.69, *p*=.000) in the alpha band. For children, none of the metrics showed significant associations with autistic trait scores. **p*<.05

## **SI7** Graph metric means for autistic and comparison groups per age group

|  | Clustering coefficient | |  | Path length | |  | Global efficiency | |  | Small Worldness | |
| --- | --- | --- | --- | --- | --- | --- | --- | --- | --- | --- | --- |
| *Children* |  | |  |  | |  |  | |  |  | |
| Frequency band/ threshold | Autistic group  mean (sd) | Comparison group  mean (sd) |  | Autistic group  mean (sd) | Comparison group  mean (sd) |  | Autistic group  mean (sd) | Comparison group  mean (sd) |  | Autistic group  mean (sd) | Comparison group  mean (sd) |
| Delta | | | | | | | | | | | |
| 0.05 | 0.73 (0.36) | 0.83 (0.28) |  | 1.12 (0.07) | 1.12 (0.09) |  | 0.89 (0.06) | 0.91 (0.06) |  | 0.65 (0.32) | 0.73 (0.24) |
| 0.08 | 0.80 (0.27) | 0.90 (0.25) |  | 1.10 (0.05) | 1.11 (0.07) |  | 0.94 (0.03) | 0.93 (0.04) |  | 0.74 (0.26) | 0.82 (0.24) |
| 0.11 | 0.82 (0.18) | 0.88 (0.19) |  | 1.08 (0.04) | 1.08 (0.04) |  | 0.95 (0.02) | 0.95 (0.02) |  | 0.76 (0.18) | 0.82 (0.18) |
| 0.13 | 0.87 (0.14) | 0.89 (0.15) |  | 1.06 (0.03) | 1.06 (0.03) |  | 0.96 (0.02) | 0.96 (0.02) |  | 0.82 (0.14) | 0.83 (0.15) |
| 0.16 | 0.89 (0.12) | 0.90 (0.12) |  | 1.05 (0.02) | 1.05 (0.02) |  | 0.97 (0.01) | 0.97 (0.01) |  | 0.84 (0.12) | 0.85 (0.12) |
| 0.19 | 0.90 (0.10) | 0.92 (0.11) |  | 1.04 (0.02) | 1.04 (0.02) |  | 0.98 (0.01) | 0.98 (0.01) |  | 0.86 (0.10) | 0.89 (0.11) |
| 0.22 | 0.91 (0.08) | 0.92 (0.11) |  | 1.03 (0.01) | 1.03 (0.01) |  | 0.99 (0.01) | 0.98 (0.01) |  | 0.89 (0.09) | 0.90 (0.11) |
| 0.24 | 0.93 (0.07) | 0.94 (0.09) |  | 1.02 (0.01) | 1.02 (0.01) |  | 0.99 (0.00) | 0.99 (0.00) |  | 0.91 (0.07) | 0.92 (0.09) |
| 0.27 | 0.94 (0.06) | 0.95 (0.08) |  | 1.01 (0.01) | 1.02 (0.01) |  | 0.99 (0.00) | 0.99 (0.00) |  | 0.93 (0.07) | 0.93 (0.08) |
| 0.30 | 0.95 (0.05) | 0.96 (0.07) |  | 1.01 (0.01) | 1.01 (0.01) |  | 1.00 (0.00) | 1.00 (0.00) |  | 0.94 (0.05) | 0.95 (0.07) |
| Theta | | | | | | | | | | | |
| 0.05 | 0.93 (0.38) | 0.99 (0.37) |  | 1.15 (0.12) | 1.13 (0.08) |  | 0.89 (0.06) | 0.91 (0.04) |  | 0.81 (0.33) | 0.87 (0.32) |
| 0.08 | 0.98 (0.33) | 0.99 (0.32) |  | 1.12 (0.07) | 1.10 (0.04) |  | 0.92 (0.04) | 0.93 (0.02) |  | 0.87 (0.29) | 0.90 (0.29) |
| 0.11 | 1.00 (0.25) | 0.96 (0.25) |  | 1.09 (0.04) | 1.08 (0.03) |  | 0.95 (0.02) | 0.94 (0.02) |  | 0.92 (0.23) | 0.89 (0.24) |
| 0.13 | 0.99 (0.20) | 0.94 (0.21) |  | 1.07 (0.03) | 1.07 (0.03) |  | 0.96 (0.02) | 0.95 (0.03) |  | 0.93 (0.19) | 0.89 (0.20) |
| 0.16 | 1.00 (0.17) | 0.96 (0.18) |  | 1.06 (0.03) | 1.07 (0.04) |  | 0.97 (0.01) | 0.97 (0.02) |  | 0.94 (0.16) | 0.91 (0.18) |
| 0.19 | 1.00 (0.14) | 0.97 (0.16) |  | 1.05 (0.02) | 1.05 (0.02) |  | 0.97 (0.01) | 0.97 (0.01) |  | 0.95 (0.14) | 0.92 (0.16) |
| 0.22 | 0.98 (0.12) | 0.97 (0.14) |  | 1.04 (0.01) | 1.04 (0.02) |  | 0.98 (0.01) | 0.98 (0.01) |  | 0.95 (0.12) | 0.94 (0.14) |
| 0.24 | 0.98 (0.09) | 0.97 (0.12) |  | 1.03 (0.01) | 1.03 (0.02) |  | 0.99 (0.01) | 0.99 (0.01) |  | 0.95 (0.10) | 0.94 (0.12) |
| 0.27 | 0.98 (0.08) | 0.97 (0.10) |  | 1.02 (0.01) | 1.02 (0.01) |  | 0.99 (0.01) | 0.99 (0.01) |  | 0.96 (0.08) | 0.96 (0.10) |
| 0.30 | 0.98 (0.07) | 0.98 (0.08) |  | 1.02 (0.01) | 1.01 (0.01) |  | 0.99 (0.00) | 0.99 (0.01) |  | 0.97 (0.07) | 0.96 (0.08) |
| Alpha | | | | | | | | | | | |
| 0.05 | 1.20 (0.37) | 1.06 (0.37) |  | 1.16 (0.10) | 1.13 (0.11) |  | 0.90 (0.06) | 0.89 (0.07) |  | 1.03 (0.29) | 0.95 (0.32) |
| 0.08 | 1.16 (0.29) | 1.05 (0.29) |  | 1.11 (0.08) | 1.12 (0.07) |  | 0.93 (0.05) | 0.93 (0.03) |  | 1.04 (0.24) | 0.94 (0.26) |
| 0.11 | 1.12 (0.24) | 1.04 (0.23) |  | 1.10 (0.07) | 1.09 (0.04) |  | 0.94 (0.03) | 0.95 (0.02) |  | 1.02 (0.23) | 0.96 (0.21) |
| 0.13 | 1.09 (0.20) | 1.03 (0.18) |  | 1.07 (0.04) | 1.07 (0.03) |  | 0.96 (0.02) | 0.96 (0.02) |  | 1.02 (0.19) | 0.97 (0.16) |
| 0.16 | 1.07 (0.16) | 1.00 (0.17) |  | 1.06 (0.03) | 1.05 (0.03) |  | 0.97 (0.02) | 0.97 (0.02) |  | 1.02 (0.16) | 0.95 (0.16) |
| 0.19 | 1.05 (0.12) | 1.00 (0.13) |  | 1.04 (0.02) | 1.04 (0.02) |  | 0.97 (0.01) | 0.98 (0.01) |  | 1.01 (0.12) | 0.96 (0.13) |
| 0.22 | 1.05 (0.11) | 1.00 (0.12) |  | 1.04 (0.02) | 1.03 (0.02) |  | 0.98 (0.01) | 0.98 (0.01) |  | 1.01 (0.11) | 0.97 (0.12) |
| 0.24 | 1.04 (0.09) | 1.01 (0.10) |  | 1.03 (0.01) | 1.02 (0.01) |  | 0.99 (0.01) | 0.99 (0.01) |  | 1.01 (0.09) | 0.98 (0.10) |
| 0.27 | 1.04 (0.07) | 1.01 (0.08) |  | 1.02 (0.01) | 1.02 (0.01) |  | 0.99 (0.00) | 0.99 (0.00) |  | 1.02 (0.07) | 0.99 (0.08) |
| 0.30 | 1.03 (0.06) | 1.01 (0.07) |  | 1.01 (0.01) | 1.01 (0.01) |  | 1.00 (0.00) | 1.00 (0.00) |  | 1.02 (0.06) | 1.00 (0.07) |
| Beta | | | | | | | | | | | |
| 0.05 | 1.03 (0.39) | 1.07 (0.43) |  | 1.15 (0.09) | 1.14 (0.11) |  | 0.89 (0.07) | 0.89 (0.06) |  | 0.90 (0.35) | 0.93 (0.34) |
| 0.08 | 1.05 (0.31) | 1.05 (0.31) |  | 1.11 (0.06) | 1.12 (0.06) |  | 0.93 (0.04) | 0.93 (0.03) |  | 0.95 (0.28) | 0.94 (0.27) |
| 0.11 | 1.07 (0.23) | 1.03 (0.25) |  | 1.09 (0.04) | 1.09 (0.04) |  | 0.95 (0.02) | 0.94 (0.02) |  | 0.98 (0.22) | 0.95 (0.22) |
| 0.13 | 1.05 (0.18) | 1.01 (0.20) |  | 1.07 (0.03) | 1.07 (0.03) |  | 0.96 (0.02) | 0.96 (0.02) |  | 0.99 (0.17) | 0.95 (0.18) |
| 0.16 | 1.03 (0.16) | 1.01 (0.17) |  | 1.05 (0.02) | 1.06 (0.02) |  | 0.97 (0.01) | 0.97 (0.01) |  | 0.98 (0.15) | 0.95 (0.16) |
| 0.19 | 1.02 (0.13) | 1.00 (0.14) |  | 1.04 (0.02) | 1.05 (0.02) |  | 0.98 (0.01) | 0.98 (0.01) |  | 0.98 (0.13) | 0.96 (0.14) |
| 0.22 | 1.02 (0.12) | 0.98 (0.13) |  | 1.03 (0.02) | 1.04 (0.02) |  | 0.98 (0.01) | 0.98 (0.01) |  | 0.98 (0.11) | 0.95 (0.13) |
| 0.24 | 1.01 (0.11) | 0.98 (0.11) |  | 1.02 (0.01) | 1.03 (0.01) |  | 0.99 (0.01) | 0.99 (0.01) |  | 0.99 (0.10) | 0.95 (0.11) |
| 0.27 | 1.00 (0.09) | 0.98 (0.09) |  | 1.02 (0.01) | 1.02 (0.01) |  | 0.99 (0.00) | 0.99 (0.00) |  | 0.99 (0.09) | 0.96 (0.09) |
| 0.30 | 1.00 (0.07) | 0.98 (0.07) |  | 1.01 (0.01) | 1.01 (0.01) |  | 1.00 (0.00) | 0.99 (0.00) |  | 0.99 (0.07) | 0.97 (0.07) |
| Adolescents |  |  |  |  |  |  |  |  |  |  |  |
| Frequency band/ threshold | Autistic group  mean (sd) | Comparison group  mean (sd) |  | Autistic group  mean (sd) | Comparison group  mean (sd) |  | Autistic group  mean (sd) | Comparison group  mean (sd) |  | Autistic group  mean (sd) | Comparison group  mean (sd) |
| Delta |  |  |  |  |  |  |  |  |  |  |  |
| 0.05 | 0.72 (0.34) | 0.72 (0.33) |  | 1.12 (0.08) | 1.11 (0.08) |  | 0.91 (0.05) | 0.90 (0.07) |  | 0.64 (0.30) | 0.65 (0.29) |
| 0.08 | 0.78 (0.28) | 0.76 (0.24) |  | 1.10 (0.05) | 1.10 (0.05) |  | 0.93 (0.03) | 0.93 (0.04 |  | 0.71 (0.26) | 0.69 (0.22) |
| 0.11 | 0.82 (0.23 | 0.81 (0.20) |  | 1.08 (0.03) | 1.09 (0.05) |  | 0.95 (0.02) | 0.95 (0.02 |  | 0.76 (0.21) | 0.75 (0.19) |
| 0.13 | 0.85 (0.19) | 0.84 (0.17) |  | 1.07 (0.02) | 1.07 (0.04) |  | 0.96 (0.02) | 0.96 (0.02) |  | 0.79 (0.18) | 0.79 (0.17) |
| 0.16 | 0.87 (0.17) | 0.86 (0.16) |  | 1.06 (0.02) | 1.06 (0.03) |  | 0.97 (0.01) | 0.97 (0.02) |  | 0.82 (0.16) | 0.82 (0.16) |
| 0.19 | 0.89 (0.14) | 0.88 (0.15) |  | 1.05 (0.02) | 1.05 (0.02) |  | 0.98 (0.01) | 0.98 (0.01) |  | 0.85 (0.14) | 0.84 (0.15) |
| 0.22 | 0.90 (0.12) | 0.90 (0.13) |  | 1.04 (0.02) | 1.04 (0.02) |  | 0.98 (0.01) | 0.98 (0.01) |  | 0.87 (0.12) | 0.87 (0.14) |
| 0.24 | 0.92 (0.10) | 0.91 (0.12 |  | 1.03 (0.01) | 1.03 (0.01) |  | 0.99 (0.01) | 0.99 (0.01) |  | 0.90 (0.11) | 0.89 (0.12) |
| 0.27 | 0.94 (0.09) | 0.92 (0.10) |  | 1.02 (0.01) | 1.02 (0.01) |  | 0.99 (0.01) | 0.99 (0.01) |  | 0.92 (0.09) | 0.91 (0.10) |
| 0.30 | 0.94 (0.08) | 0.94 (0.08) |  | 1.01 (0.01) | 1.01 (0.01) |  | 0.99 (0.00) | 0.99 (0.00) |  | 0.93 (0.08) | 0.92 (0.09) |
| Theta |  |  |  |  |  |  |  |  |  |  |  |
| 0.05 | 0.90 (0.46) | 0.94 (0.36) |  | 1.13 (0.09) | 1.13 (0.10) |  | 0.89 (0.07) | 0.88 (0.09) |  | 0.79 (0.40) | 0.83 (0.32) |
| 0.08 | 0.93 (0.31) | 0.97 (0.29) |  | 1.12 (0.07) | 1.13 (0.08) |  | 0.92 (0.05) | 0.92 (0.05) |  | 0.83 (0.27) | 0.86 (0.27) |
| 0.11 | 0.94 (0.25) | 0.95 (0.23) |  | 1.10 (0.05) | 1.10 (0.06) |  | 0.94 (0.02) | 0.94 (0.03) |  | 0.86 (0.23) | 0.87 (0.21) |
| 0.13 | 0.94 (0.20) | 0.95 (0.21) |  | 1.08 (0.04) | 1.09 (0.04) |  | 0.95 (0.02) | 0.95 (0.02) |  | 0.88 (0.19) | 0.88 (0.19) |
| 0.16 | 0.96 (0.17) | 0.96 (0.19) |  | 1.07 (0.03) | 1.07 (0.03) |  | 0.96 (0.01) | 0.96 (0.02) |  | 0.90 (0.17) | 0.89 (0.18) |
| 0.19 | 0.96 (0.15) | 0.96 (0.17) |  | 1.05 (0.02) | 1.06 (0.03) |  | 0.97 (0.01) | 0.97 (0.01) |  | 0.91 (0.14 | 0.91 (0.16) |
| 0.22 | 0.97 (0.13) | 0.96 (0.15) |  | 1.04 (0.02) | 1.05 (0.02) |  | 0.98 (0.01) | 0.97 (0.01) |  | 0.93 (0.13) | 0.92 (0.14) |
| 0.24 | 0.97 (0.11) | 0.96 (0.12) |  | 1.03 (0.02) | 1.04 (0.02) |  | 0.98 (0.01 | 0.98 (0.01) |  | 0.94 (0.11) | 0.93 (0.13) |
| 0.27 | 0.97 (0.09) | 0.96 (0.11) |  | 1.03 (0.01) | 1.03 (0.01) |  | 0.99 (0.01) | 0.99 (0.01) |  | 0.94 (0.09) | 0.94 (0.11) |
| 0.30 | 0.97 (0.08) | 0.96 (0.09) |  | 1.02 (0.01) | 1.02 (0.01) |  | 0.99 (0.00) | 0.99 (0.01) |  | 0.95 (0.08) | 0.95 (0.10) |
| Alpha |  |  |  |  |  |  |  |  |  |  |  |
| 0.05 | 1.02 (0.40) | 1.01 (0.36) |  | 1.14 (0.11) | 1.13 (0.11) |  | 0.91 (0.06) | 0.90 (0.06) |  | 0.90 (0.32) | 0.89 (0.27) |
| 0.08 | 1.03 (0.33) | 1.00 (0.33) |  | 1.11 (0.07) | 1.11 (0.07) |  | 0.93 (0.04) | 0.93 (0.04) |  | 0.92 (0.27) | 0.89 (0.26) |
| 0.11 | 1.04 (0.27) | 1.00 (0.30) |  | 1.09 (0.05) | 1.09 (0.05) |  | 0.95 (0.03) | 0.94 (0.03) |  | 0.95 (0.23) | 0.91 (0.26) |
| 0.13 | 1.02 (0.23) | 0.98 (0.24) |  | 1.08 (0.04) | 1.07 (0.04) |  | 0.96 (0.02) | 0.96 (0.02) |  | 0.94 (0.20) | 0.91 (0.21) |
| 0.16 | 1.02 (0.19) | 0.99 (0.21) |  | 1.07 (0.03) | 1.06 (0.03) |  | 0.96 (0.02) | 0.97 (0.02) |  | 0.95 (0.17) | 0.93 (0.19) |
| 0.19 | 1.00 (0.16) | 0.99 (0.17) |  | 1.06 (0.02) | 1.05 (0.02) |  | 0.97 (0.01) | 0.97 (0.01) |  | 0.95 (0.15) | 0.95 (0.16) |
| 0.22 | 1.00 (0.14) | 1.00 (0.14) |  | 1.04 (0.02 | 1.04 (0.02) |  | 0.98 (0.01) | 0.98 (0.01) |  | 0.96 (0.14 | 0.96 (0.13) |
| 0.24 | 0.99 (0.12) | 0.99 (0.12) |  | 1.03 (0.01) | 1.03 (0.02) |  | 0.98 (0.01) | 0.99 (0.01) |  | 0.96 (0.12) | 0.96 (0.12) |
| 0.27 | 0.99 (0.10) | 0.99 (0.11) |  | 1.03 (0.01) | 1.02 (0.01) |  | 0.99 (0.01) | 0.99 (0.01) |  | 0.97 (0.10) | 0.97 (0.11) |
| 0.30 | 0.99 (0.09) | 0.99 (0.09) |  | 1.02 (0.01) | 1.02 (0.01) |  | 0.99 (0.00) | 0.99 (0.00) |  | 0.98 (0.09) | 0.98 (0.09) |
| Beta |  |  |  |  |  |  |  |  |  |  |  |
| 0.05 | 1.07 (0.41) | 1.11 (0.46) |  | 1.14 (0.10) | 1.18 (0.11) |  | 0.90 (0.06) | 0.87 (0.07) |  | 0.94 (0.34) | 0.94 (0.39) |
| 0.08 | 1.04 (0.33) | 1.08 (0.33) |  | 1.10 (0.06) | 1.13 (0.06) |  | 0.92 (0.05) | 0.92 (0.05) |  | 0.93 (0.29) | 0.96 (0.28) |
| 0.11 | 1.03 (0.26) | 1.06 (0.25) |  | 1.09 (0.04) | 1.11 (0.08) |  | 0.94 (0.03) | 0.94 (0.03) |  | 0.95 (0.24) | 0.96 (0.24) |
| 0.13 | 0.99 (0.21) | 1.03 (0.19) |  | 1.08 (0.03) | 1.09 (0.04) |  | 0.95 (0.02) | 0.95 (0.02) |  | 0.93 (0.20) | 0.95 (0.18) |
| 0.16 | 1.00 (0.18) | 1.03 (0.17) |  | 1.06 (0.03) | 1.07 (0.03) |  | 0.96 (0.01 | 0.96 (0.01) |  | 0.94 (0.18) | 0.97 (0.17) |
| 0.19 | 1.00 (0.16) | 1.02 (0.15) |  | 1.05 (0.02) | 1.05 (0.02) |  | 0.97 (0.01) | 0.97 (0.01) |  | 0.95 (0.15) | 0.97 (0.15) |
| 0.22 | 1.00 (0.14) | 1.01 (0.13) |  | 1.04 (0.02) | 1.04 (0.02) |  | 0.98 (0.01) | 0.98 (0.01) |  | 0.96 (0.14) | 0.97 (0.13) |
| 0.24 | 1.00 (0.11) | 1.01 (0.10) |  | 1.03 (0.01) | 1.03 (0.01) |  | 0.99 (0.01) | 0.99 (0.01) |  | 0.97 (0.11) | 0.98 (0.10) |
| 0.27 | 1.00 (0.09) | 1.01 (0.08) |  | 1.02 (0.01) | 1.02 (0.01) |  | 0.99 (0.01) | 0.99 (0.00) |  | 0.98 (0.10) | 0.99 (0.08) |
| 0.30 | 1.00 (0.08) | 1.01 (0.07) |  | 1.02 (0.01) | 1.01 (0.01) |  | 0.99 (0.00) | 0.99 (0.00) |  | 0.98 (0.08) | 0.99 (0.08) |
| *Adults* |  |  |  |  |  |  |  |  |  |  |  |
| Frequency band/ threshold | Autistic group  mean (sd) | Comparison group  mean (sd) |  | Autistic group  mean (sd) | Comparison group  mean (sd) |  | Autistic group  mean (sd) | Comparison group  mean (sd) |  | Autistic group  mean (sd) | Comparison group  mean (sd) |
| Delta | | | | | | | | | | | |
| 0.05 | 0.75 (0.38) | 0.66 (0.35) |  | 1.16 (0.14) | 1.11 (0.08) |  | 0.90 (0.06) | 0.91 (0.07) |  | 0.65 (0.32) | 0.60 (0.30) |
| 0.08 | 0.78 (0.29) | 0.73 (0.26) |  | 1.10 (0.05) | 1.10 (0.06) |  | 0.93 (0.04) | 0.93 (0.03) |  | 0.70 (0.26) | 0.66 (0.23) |
| 0.11 | 0.81 (0.24) | 0.78 (0.22) |  | 1.08 (0.04) | 1.08 (0.04) |  | 0.94 (0.03) | 0.95 (0.03) |  | 0.75 (0.22) | 0.72 (0.20) |
| 0.13 | 0.84 (0.19) | 0.79 (0.20) |  | 1.07 (0.04) | 1.07 (0.04) |  | 0.96 (0.02) | 0.96 (0.02) |  | 0.78 (0.19) | 0.74 (0.20) |
| 0.16 | 0.86 (0.16) | 0.80 (0.17) |  | 1.06 (0.03) | 1.06 (0.03) |  | 0.97 (0.02) | 0.97 (0.02) |  | 0.81 (0.15) | 0.76 (0.17) |
| 0.19 | 0.88 (0.14) | 0.83 (0.15) |  | 1.05 (0.02) | 1.05 (0.02) |  | 0.98 (0.01) | 0.97 (0.01) |  | 0.84 (0.14) | 0.79 (0.15) |
| 0.22 | 0.90 (0.13) | 0.85 (0.13) |  | 1.04 (0.02) | 1.04 (0.02) |  | 0.98 (0.01) | 0.98 (0.01) |  | 0.86 (0.13) | 0.82 (0.13) |
| 0.24 | 0.92 (0.11) | 0.87 (0.11) |  | 1.03 (0.02) | 1.03 (0.02) |  | 0.99 (0.01) | 0.99 (0.01) |  | 0.89 (0.11) | 0.85 (0.11) |
| 0.27 | 0.93 (0.10) | 0.90 (0.10) |  | 1.02 (0.01) | 1.02 (0.01) |  | 0.99 (0.01) | 0.99 (0.01) |  | 0.91 (0.10) | 0.88 (0.10) |
| 0.30 | 0.94 (0.08) | 0.91 (0.08) |  | 1.01 (0.01) | 1.02 (0.01) |  | 0.99 (0.00) | 0.99 (0.00) |  | 0.92 (0.08) | 0.90 (0.09) |
| Theta | | | | | | | | | | | |
| 0.05 | 0.75 (0.28) | 0.81 (0.37) |  | 1.13 (0.08) | 1.14 (0.12) |  | 0.91 (0.04) | 0.88 (0.07) |  | 0.66 (0.25) | 0.71 (0.32) |
| 0.08 | 0.81 (0.23) | 0.85 (0.29) |  | 1.10 (0.05) | 1.12 (0.08) |  | 0.93 (0.03) | 0.92 (0.04) |  | 0.73 (0.21) | 0.76 (0.26) |
| 0.11 | 0.83 (0.19) | 0.88 (0.25) |  | 1.09 (0.04) | 1.10 (0.06) |  | 0.94 (0.03) | 0.94 (0.04) |  | 0.76 (0.18) | 0.80 (0.23) |
| 0.13 | 0.84 (0.17) | 0.90 (0.21) |  | 1.08 (0.03) | 1.09 (0.06) |  | 0.95 (0.02) | 0.95 (0.03) |  | 0.78 (0.16) | 0.83 (0.20) |
| 0.16 | 0.88 (0.16) | 0.92 (0.18) |  | 1.07 (0.04) | 1.07 (0.04) |  | 0.96 (0.02) | 0.96 (0.02) |  | 0.83 (0.16) | 0.86 (0.18) |
| 0.19 | 0.90 (0.15) | 0.91 (0.15) |  | 1.06 (0.03) | 1.06 (0.03) |  | 0.97 (0.01) | 0.97 (0.02) |  | 0.85 (0.15) | 0.86 (0.16) |
| 0.22 | 0.91 (0.13) | 0.92 (0.14) |  | 1.05 (0.03) | 1.05 (0.03) |  | 0.98 (0.01) | 0.98 (0.01) |  | 0.87 (0.14) | 0.87 (0.14) |
| 0.24 | 0.92 (0.12) | 0.92 (0.12) |  | 1.04 (0.02) | 1.04 (0.02) |  | 0.98 (0.01) | 0.98 (0.01) |  | 0.88 (0.12) | 0.89 (0.13) |
| 0.27 | 0.93 (0.10) | 0.93 (0.11) |  | 1.03 (0.02) | 1.03 (0.02) |  | 0.99 (0.01) | 0.99 (0.01) |  | 0.90 (0.11) | 0.91 (0.11) |
| 0.30 | 0.94 (0.09) | 0.93 (0.10) |  | 1.02 (0.01) | 1.02 (0.02) |  | 0.99 (0.01) | 0.99 (0.01) |  | 0.92 (0.10) | 0.92 (0.10) |
| Alpha | | | | | | | | | | | |
| 0.05 | 0.88 (0.33) | 1.03 (0.32) |  | 1.11 (0.09) | 1.13 (0.10) |  | 0.91 (0.07) | 0.91 (0.06) |  | 0.79 (0.30) | 0.91 (0.29) |
| 0.08 | 0.90 (0.24) | 1.01 (0.29) |  | 1.11 (0.06) | 1.10 (0.06) |  | 0.93 (0.04) | 0.93 (0.03) |  | 0.81 (0.22) | 0.91 (0.26) |
| 0.11 | 0.92 (0.22) | 1.00 (0.26) |  | 1.09 (0.05) | 1.08 (0.04) |  | 0.94 (0.03) | 0.94 (0.03) |  | 0.84 (0.21) | 0.92 (0.23) |
| 0.13 | 0.93 (0.19) | 1.00 (0.23) |  | 1.08 (0.05) | 1.08 (0.03) |  | 0.96 (0.02) | 0.95 (0.02) |  | 0.87 (0.18) | 0.93 (0.21) |
| 0.16 | 0.94 (0.17) | 0.98 (0.20) |  | 1.06 (0.04) | 1.07 (0.03) |  | 0.96 (0.02) | 0.96 (0.02) |  | 0.89 (0.16) | 0.92 (0.18) |
| 0.19 | 0.96 (0.14) | 0.98 (0.18) |  | 1.05 (0.03) | 1.06 (0.04) |  | 0.97 (0.01) | 0.97 (0.02) |  | 0.91 (0.14) | 0.92 (0.17) |
| 0.22 | 0.96 (0.13) | 0.97 (0.15) |  | 1.04 (0.03) | 1.05 (0.03) |  | 0.98 (0.01) | 0.98 (0.01) |  | 0.92 (0.13) | 0.93 (0.15) |
| 0.24 | 0.96 (0.12) | 0.97 (0.14) |  | 1.04 (0.02) | 1.04 (0.02) |  | 0.98 (0.01) | 0.98 (0.01) |  | 0.93 (0.12) | 0.93 (0.13) |
| 0.27 | 0.96 (0.10) | 0.97 (0.12) |  | 1.03 (0.02) | 1.03 (0.02) |  | 0.99 (0.01) | 0.99 (0.01) |  | 0.94 (0.10) | 0.94 (0.12) |
| 0.30 | 0.97 (0.09) | 0.97 (0.11) |  | 1.02 (0.01) | 1.02 (0.01) |  | 0.99 (0.01) | 0.99 (0.01) |  | 0.95 (0.09) | 0.95 (0.11) |
| Beta | | | | | | | | | | | |
| 0.05 | 0.92 (0.36) | 0.98 (0.40) |  | 1.14 (0.10) | 1.14 (0.11) |  | 0.89 (0.07) | 0.89 (0.09) |  | 0.81 (0.31) | 0.87 (0.36) |
| 0.08 | 0.94 (0.27) | 1.04 (0.29) |  | 1.12 (0.09) | 1.14 (0.08) |  | 0.93 (0.04) | 0.92 (0.04) |  | 0.84 (0.25) | 0.92 (0.27) |
| 0.11 | 0.96 (0.25) | 1.02 (0.24) |  | 1.11 (0.06) | 1.10 (0.05) |  | 0.94 (0.03) | 0.94 (0.03) |  | 0.87 (0.24) | 0.93 (0.23) |
| 0.13 | 0.97 (0.21) | 1.00 (0.20) |  | 1.08 (0.04) | 1.09 (0.04) |  | 0.95 (0.02) | 0.95 (0.02) |  | 0.89 (0.20) | 0.92 (0.19) |
| 0.16 | 0.96 (0.18) | 1.01 (0.17) |  | 1.07 (0.03) | 1.07 (0.03) |  | 0.96 (0.02) | 0.96 (0.01) |  | 0.90 (0.17) | 0.94 (0.17) |
| 0.19 | 0.96 (0.16) | 1.00 (0.14) |  | 1.06 (0.03) | 1.06 (0.02) |  | 0.97 (0.01) | 0.97 (0.01) |  | 0.91 (0.16) | 0.95 (0.14) |
| 0.22 | 0.97 (0.13) | 1.00 (0.12) |  | 1.05 (0.02) | 1.04 (0.02) |  | 0.98 (0.01) | 0.98 (0.01) |  | 0.93 (0.13) | 0.96 (0.12) |
| 0.24 | 0.98 (0.11) | 1.00 (0.11) |  | 1.03 (0.02) | 1.03 (0.02) |  | 0.98 (0.01) | 0.99 (0.01) |  | 0.95 (0.11) | 0.97 (0.11) |
| 0.27 | 0.98 (0.10) | 1.00 (0.10) |  | 1.03 (0.01) | 1.02 (0.01) |  | 0.99 (0.01) | 0.99 (0.01) |  | 0.95 (0.10) | 0.98 (0.10) |
| 0.30 | 0.98 (0.08) | 1.00 (0.09) |  | 1.02 (0.01) | 1.01 (0.01) |  | 0.99 (0.00) | 0.99 (0.00) |  | 0.96 (0.09) | 0.99 (0.09) |

## **SI8** Predicting social cognition by graph metrics across autistic and non-autistic adults

| *Adults* |  |  |  |  |  |  |  |  |  |  |  |  |  |  |  |
| --- | --- | --- | --- | --- | --- | --- | --- | --- | --- | --- | --- | --- | --- | --- | --- |
|  | EQ (n=118) | | |  | RMET (n=124) | | |  | AS ToM  (n=115) | | |  | AS Rand (n=115) | | |
|  | Beta | *p* | *R^2^* |  | Beta | *p* | *R^2^* |  | Beta | *p* | *R^2^* |  | Beta | *p* | *R^2^* |
| Clustering | 1.53 | .53 | <.01 |  | 4.32 | .23 | <.01 |  | -2.09e-01 | .68 | <.01 |  | -4.36e-01 | .23 | .01 |
| Sex | 1.20 | .49 | <.01 |  | -5.47e-01 | .83 | <.01 |  | 4.19e-01 | .25 | .01 |  | -1.89e-01 | .46 | <.01 |
| Age^2^ | -9.19e-04 | .85 | <.01 |  | 141e-04 | .98 | <.01 |  | -1.70e-03 | .10 | .02 |  | -8.98e-04 | .22 | .01 |
| IQ | 8.89e-02 | .17 | .02 |  | 2.77e-01 | .003* | <.01 |  | 2.02e-02 | .13 | .02 |  | 1.45e-02 | .13 | .02 |
| Site | 5.17 | .02* | .08 |  | -1.55 | .63 | <.01 |  | -1.28e-01 | .78 | <.01 |  | -1.80e-01 | .58 | .15 |
| Clustering*SRS | -4.26e-02 | .31 | <.01 |  | -3.58e-02 | .73 | <.01 |  | -2.65e-03 | .86 | <.01 |  | -1.60e-03 | .88 | <.01 |
|  | EQ (n=118) | | |  | RMET (n=124) | | |  | AS ToM  (n=115) | | |  | AS Rand (n=115) | | |
|  | Beta | *p* | *R^2^* |  | Beta | *p* | *R^2^* |  | Beta | *p* | *R^2^* |  | Beta | *p* | *R^2^* |
| Small-worldness | 4.32 | .20 | <.01 |  | 3.47 | .47 | <.01 |  | -3.21e-01 | .64 | <.01 |  | -1.10 | .03* | .04 |
| Sex | 1.28 | .46 | <.01 |  | -6.59-01 | .79 | <.01 |  | 4.15e-01 | .25 | .01 |  | -2.24e-01 | .38 | <.01 |
| Age^2^ | -3.97e04 | .94 | <.01 |  | 2.97e-04 | .96 | <.01 |  | -1.73e-03 | .10 | .02 |  | -1.04e-03 | .15 | .02 |
| IQ | 8.97e-02 | .16 | <.01 |  | 2.90e-01 | .002* | <.01 |  | 1.97e-02 | .14 | .02 |  | 1.40e-02 | .13 | .02 |
| Site | 5.28 | .01* | <.01 |  | -1.83 | .57 | <.01 |  | -1.26e-01 | .78 | <.01 |  | -1.93e-02 | .55 | .16 |
| Small-worldness*SRS | -6.24e-02 | .27 | .01 |  | -5.26e-02 | .70 | <.01 |  | 3.26e-02 | .11 | .02 |  | 1.94e-03 | .89. | <.01 |

*R^2^* = partial *R^2^*, site = UMCU-KLC contrast The model predicting EQ by clustering was not significant (R2=.10, *R^2^*_adjusted_ =.04; F(8,109)=1.59, *p=*.14). With the clustering*SRS-2 interaction, the model explained 72% of variance (*R^2^*_adjusted_=.69; F(10,107)=27.63; *p*<.001. The model predicting EQ by small-worldness was not significant (*R2*=.11; *R^2^*_adjusted_=.09; F(8,109)=1.77; *p=*.09). With the small-worldness*SRS-2 interaction, the model explained 72% of variance (*R^2^*_adjusted_=.69; F(10,107)=27.53; *p*<.001). The model predicting RMET by clustering was not significant (R2=.11; *R^2^*_adjusted_=.05; F(8,115); *p*=.07). With the clustering*SRS-2 interaction the model explained 15% of variance (*R^2^*_adjusted_=.08; F(10,113); *p=*.04). The model predicting RMET by small-worldness was not significant (R2=0.11; *R^2^*_adjusted_=.05; F(8,115); *p=*.10). With the small-worldness*SRS-2 interaction the model predicted 15% of variance (*R^2^*_adjusted_=.07; F(10,113); *p=*.04). The model predicting animated shape ToM scores by clustering was not significant (R2=.069; *R^2^*_adjusted_=.003; F(8,113)=1.04; *p=*.41). The model with the clustering*SRS-2 interaction was not significant (R2=0.05, *R^2^*_adjusted_<0.001; F(9,112)=0.72; *p=*.69). The model predicting animated shape ToM scores by small-worldness was not significant (R2=0.07; *R^2^*_adjusted_=0.003; F(8,113)=1.05; *p=*.41). The model with the small-worldness*SRS-2 interaction was not significant (R2=0.08; *R^2^*_adjusted_=.003; F(9,112)=1.03; *p=*.042). The model predicting animated shape random scores by clustering explained 19% of variance (*R^2^*_adjusted_=0.13; F(8,113)=3.22; *p*=.002). The model with the clustering*SRS-2 interaction explained 19% of variance (*R^2^*_adjusted_=0.11; F(10,111); *p*=.008). The model predicting animated shape random scores by small-worldness explained 21% (*R^2^*_adjusted_=.15; F(8,113)=3.78; *p*<.001). The model with the small-worldness*SRS-2 interaction explained 21% variance (*R^2^*_adjusted_=0.14; F(10,111)=2.98; *p*=.002). *p*<.05

## **SI9** Associations between global efficiency and social cognition measures across autistic and non-autistic adults

SRS-2 = Social Responsiveness Scale-2 raw scores. The upper row shows the outcome variables EQ total scores, RMET % correct, and AS ToM scores, respectively, regressed on clustering or small-worldness in the alpha band. No measures reached significance. The second row shows visualizations of the added interaction term (E*SRS) for each model. No interaction term was significant. All models were adjusted for age, sex, IQ and site. *p*<.05


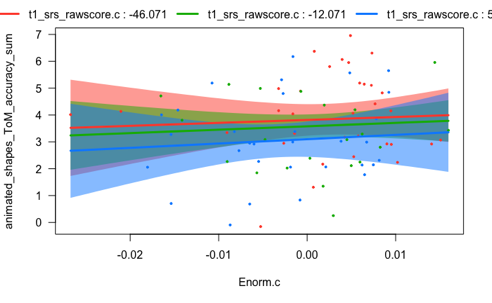

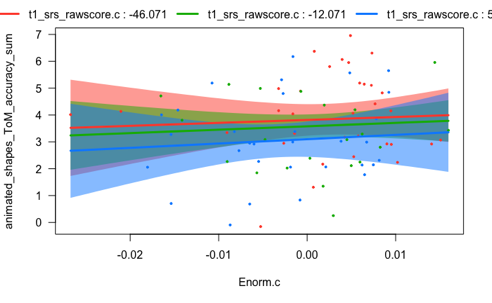

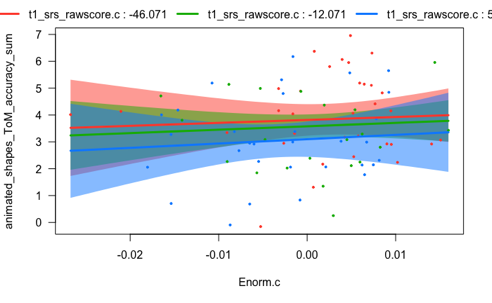


SRS-2 scores 10^th^ quantile

SRS-2 scores 50^th^ quantile

SRS-2 scores 90^th^ quantile


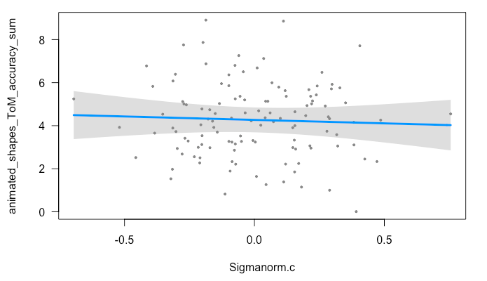


Animated Shapes ToM accuracy scores


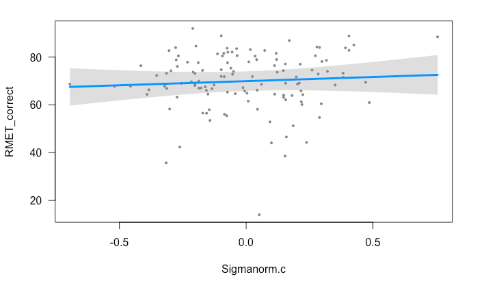


RMET % correct


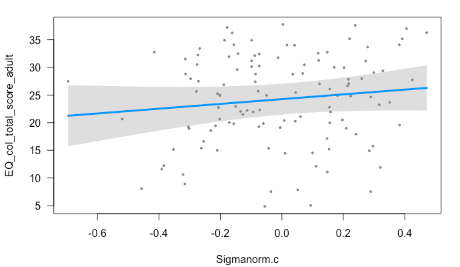


EQ total scores


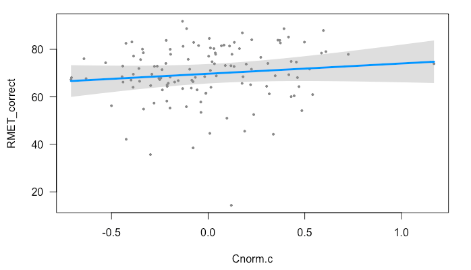


RMET % correct


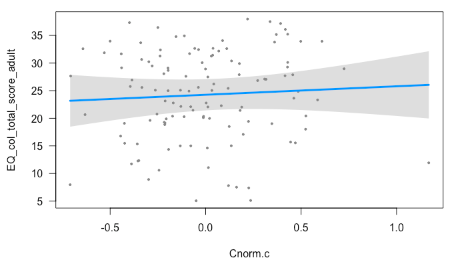


EQ total scores


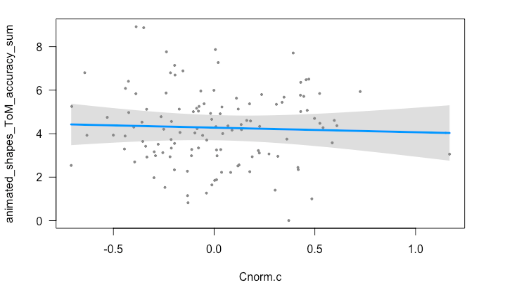


Animated Shapes ToM accuracy scores


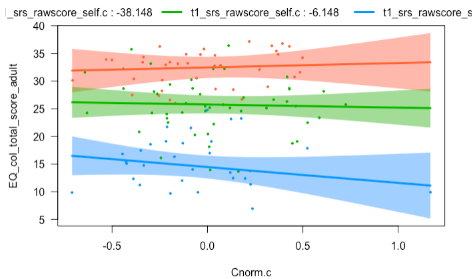


EQ total scores

Clustering


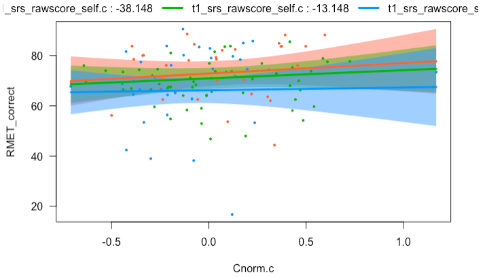


RMET % correct

Clustering


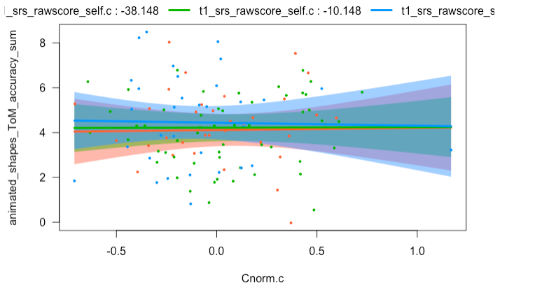


Animated Shapes ToM accuracy scores

Clustering


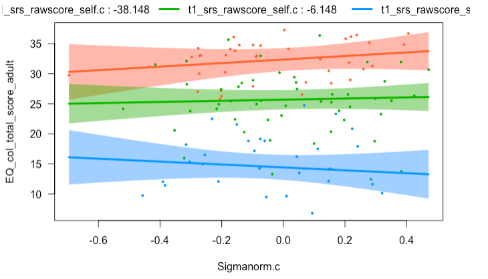


EQ total scores

Small-worldness


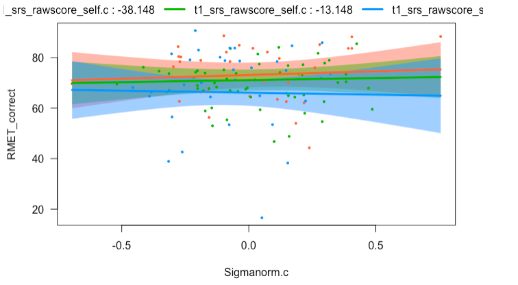


RMET % correct

Small-worldness


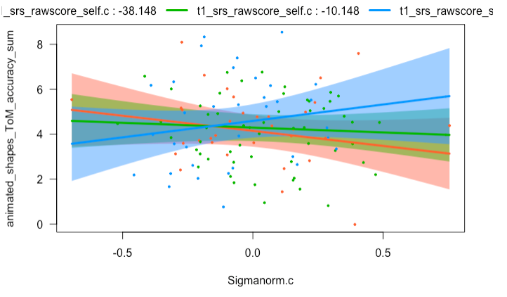


Animated Shapes ToM accuracy scores

Small-worldness

## **SI10** Bivariate correlations between all variables across autistic and non-autistic groups for each age group

### Children

| *p values* | | | | | | | |
| --- | --- | --- | --- | --- | --- | --- | --- |
| *Correlation coefficient* (Pearson) | **SRS-2** | .94 | .35 | <.0001 | .22 | .39 | .50 |
|  | 0.01 | **Age^2^** | .35 | .90 | .03 | .52 | .17 |
|  | -0.10 | -0.10 | **IQ** | .14 | .02 | .37 | .38 |
|  | -0.85**** | 0.01 | 0.16 | **EQ** | .34 | .27 | .27 |
|  | -0.14 | 0.23* | 0.26* | 0.11 | **RMET** | .42 | .14 |
|  | -0.09 | 0.07 | 0.10 | 0.13 | 0.09 | **AS ToM** | .02 |
|  | -0.07 | 0.15 | -0.10 | 0.13 | -0.17 | 0.25* | **AS rand** |

### Adolescents

| *p values* | | | | | | | | |
| --- | --- | --- | --- | --- | --- | --- | --- | --- |
| *Correlation coefficient (Pearson)* | **SRS-2** | .04 | <.001 | .054 | <.0001 | <.01 | <.01 | .80 |
|  | -0.19* | **Age^2^** | .34 | .07 | .12 | <.01 | .48 | .06 |
|  | -0.23** | 0.09 | **IQ** | .76 | .55 | <.01 | <.001 | .65 |
|  | -0.17 | -0.16 | 0.03 | **E** | .23 | .14 | .28 | .19 |
|  | -0.88**** | 0.15 | 0.06 | 0.12 | **EQ** | .01 | .01 | .91 |
|  | -0.27** | 0.24** | 0.26** | 0.14 | 0.24* | **RMET** | .09 | .75 |
|  | -0.26** | 0.07 | 0.33*** | 0.11 | 0.27* | 0.18 | **AS ToM** | .06 |
|  | -0.03 | 0.06 | 0.04 | -0.13 | 0.01 | 0.03 | 0.18 | **AS rand** |

### Adults

| *p values* | | | | | | | | | |
| --- | --- | --- | --- | --- | --- | --- | --- | --- | --- |
| *Correlation coefficient (Pearson)* | **SRS-2** | .64 | .14 | .34 | .44 | <.0001 | .01 | .52 | .43 |
|  | 0.04 | **Age^2^** | .93 | .02 | .02 | .72 | .73 | .05 | .04 |
|  | -0.13 | -0.01 | **IQ** | .26 | .32 | .08 | <.01 | .12 | .45 |
|  | -0.08 | -0.20* | 0.10 | **C** | <.0001 | .44 | .54 | .72 | .78 |
|  | -0.07 | -0.21* | 0.09 | 0.99**** | **SW** | .54 | .50 | .81 | .83 |
|  | -0.83**** | 0.03 | 0.16 | 0.07 | 0.06 | **EQ** | .01 | .54 | .06 |
|  | -0.23* | -0.03 | 0.29** | -0.06 | -0.06 | 0.24* | **RMET** | .17 | .90 |
|  | 0.06 | -0.18 | 0.14 | -0.03 | -0.02 | 0.06 | 0.13 | **AS ToM** | .01 |
|  | 0.07 | -0.18* | 0.07 | -0.03 | -0.02 | -0.17 | 0.01 | 0.24** | **AS rand** |

Note: Bivariate correlations between all variables of interest across autistic and non-autistic groups together per age group. Upper diagonal displays correlation coefficients, while the lower diagonal displays p-values. SRS-2 = Social Responsiveness Scale second edition; Age^2^ = age squared; IQ = Intelligence Coefficient; E = global efficiency in the alpha band threshold 0.22; C = clustering in the alpha band threshold 0.05; SW = small-world coefficient in the alpha band threshold 0.08; EQ = empathy quotient collapsed total scores; RMET = Reading the Mind in the Eyes Task percentage correct; AS ToM = sum correct on the theory of mind condition of the Animated Shapes task; AS Rand = sum correct on the random condition of the Animated Shapes task. ^****^ = *p*<.0001, ^***^ = *p*<.001, ^**^ = *p*<.01, ^*^ = *p*<.05.

Note: Bivariate correlations between all variables of interest across autistic and non-autistic groups together per age group. Upper diagonal displays correlation coefficients, while the lower diagonal displays p-values. SRS-2 = Social Responsiveness Scale second edition; Age^2^ = age squared; IQ = Intelligence Coefficient; E = global efficiency in the alpha band threshold 0.22; C = clustering in the alpha band threshold 0.05; SW = small-world coefficient in the alpha band threshold 0.08; EQ = empathy quotient collapsed total scores; RMET = Reading the Mind in the Eyes Task percentage correct; AS ToM = sum correct on the theory of mind condition of the Animated Shapes task; AS Rand = sum correct on the random condition of the Animated Shapes task. ^****^ = *p*<.0001, ^***^ = *p*<.001, ^**^ = *p*<.01, ^*^ = *p*<.05.

Note: Bivariate correlations between all variables of interest across autistic and non-autistic groups together per age group. Upper diagonal displays correlation coefficients, while the lower diagonal displays p-values. SRS-2 = Social Responsiveness Scale second edition; Age^2^ = age squared; IQ = Intelligence Coefficient; E = global efficiency in the alpha band threshold 0.22; C = clustering in the alpha band threshold 0.05; SW = small-world coefficient in the alpha band threshold 0.08; EQ = empathy quotient collapsed total scores; RMET = Reading the Mind in the Eyes Task percentage correct; AS ToM = sum correct on the theory of mind condition of the Animated Shapes task; AS Rand = sum correct on the random condition of the Animated Shapes task. ^****^ = *p*<.0001, ^***^ = *p*<.001, ^**^ = *p*<.01, ^*^ = *p*<.05.

**SI11** EEG quality control metrcis per site

| Children | CIMH  (n = 6) |  | KCL  (n = 25) |  | RUNMC  (n = 47) |  | UCBM  (n = 0) |  | UMCU  (n = 14) |
| --- | --- | --- | --- | --- | --- | --- | --- | --- | --- |
| Number of interpolated channels | 1.17 (0.41) |  | 4.32 (1.86) |  | 3.00 (1.37) |  | NA |  | 2.00 (1.92) |
| Number of rejected ICs | 10.8 (3.97) |  | 10.1 (3.77) |  | 12.5 (4.52) |  | NA |  | 9.86 (4.29) |
| Number of clean trials | 20.5 (5.68) |  | 17.7 (4.24) |  | 18.4 (4.68) |  | NA |  | 19.7 (4.50) |
| Adolescents | CIMH  (n = 26) |  | KCL  (n = 37) |  | RUNMC  (n = 46) |  | UCBM  (n = 0) |  | UMCU  (n =15 ) |
| Number of interpolated channels | 1.23 (0.51) |  | 3.46 (1.94) |  | 2.78 (1.21) |  | NA |  | 2.27 (1.62) |
| Number of rejected ICs | 11.0 (3.96) |  | 9.62 (3.97) |  | 11.3 (4.53) |  | NA |  | 9.60 (2.90) |
| Number of clean trials | 23.2 (3.84) |  | 20.0 (4.64) |  | 22.4 (4.10) |  | NA |  | 22.5 (3.18) |
| Adults | CIMH  (n = 7) |  | KCL  (n = 40) |  | RUNMC  (n = 29) |  | UCBM  (n = 26) |  | UMCU  (n = 26) |
| Number of interpolated channels | 1.29 (0.49) |  | 3.00 (1.28) |  | 2.66 (1.04) |  | 3.46 (2.70) |  | 2.58 (2.00) |
| Number of rejected ICs | 9.86 (5.05) |  | 9.80 (4.22) |  | 11.0 (4.77) |  | 9.50 (3.92) |  | 9.65 (3.45) |
| Number of clean trials | 22.7 (3.64) |  | 21.8 (4.40) |  | 24.1 (3.13) |  | 23.5 (3.60) |  | 19.8 (4.08) |
